# Synthesis and characterization of polysaccharide-cryogel and  its application to the electrochemical detection of DNA

**DOI:** 10.1007/s00604-024-06550-7

**Published:** 2024-08-01

**Authors:** Nilay Tunca, Meltem Maral, Esma Yildiz, Sultan Butun Sengel, Arzum Erdem

**Affiliations:** 1 The Institute of Natural and Applied Sciences, Biomedical Technologies Department, Ege University, Bornova, 35100 Izmir Turkey; 2 Analytical Chemistry Department, Faculty of Pharmacy, Ege University, Bornova, 35100 Izmir Turkey; 3 Faculty of Engineering and Architecture, Department of Biomedical Engineering, Eskisehir Osmangazi University, 26480 Eskisehir, Turkey

**Keywords:** Polypyrrole, Cryogel modified electrode; Graphite pencil electrode, Polysaccharide cryogel, Electrochemical DNA biosensor; Cyclic voltammetry

## Abstract

The main goal of our study is to demonstrate the applicability of the PPy-cryogel-modified electrodes for electrochemical detection of DNA. First, a polysaccharide-based cryogel was synthesized. This cryogel was then used as a template for chemical polypyrrole synthesis. This prepared polysaccharide-based conductive cryogel was used for electrochemical biosensing on DNA. Carrageenan (CG) and sodium alginate (SA) polysaccharides, which stand out as biocompatible materials, were used in cryogel synthesis. Electron transfer was accelerated by polypyrrole (PPy) synthesized in cryogel networks. A 2B pencil graphite electrode with a diameter of 2.00 mm was used as a working electrode. The prepared polysaccharide solution was dropped onto a working electrode as a support material to improve the immobilization capacity of biomolecules and frozen to complete the cryogelation step. PPy synthesis was performed on the electrodes whose cryogelation process was completed. In addition, the structures of cryogels synthesized on the electrode surface were characterized by thermogravimetric analysis (TGA), Fourier transform infrared spectroscopy (FTIR), and scanning electron microscopy (SEM). Surface characterization of the modified electrodes was performed by energy-dispersive X-ray spectroscopy (EDX) analysis. Electrochemical determination of fish sperm DNA (fsDNA) was performed using a PPy-cryogel-modified electrode. The use of a porous 3D cryogel intermediate material enhanced the signal by providing a large surface area for the synthesis of PPy and increasing the biomolecule immobilization capacity. The detection limit was 0.98 µg mL^−1^ in the fsDNA concentration range 2.5–20 µg mL^−1^. The sensitivity of the DNA biosensor was estimated to 14.8 µA mM^−1^ cm^−2^. The stability of the biosensor under certain storage conditions was examined and observed to remain 66.95% up to 45 days.

**Graphical Abstract:**

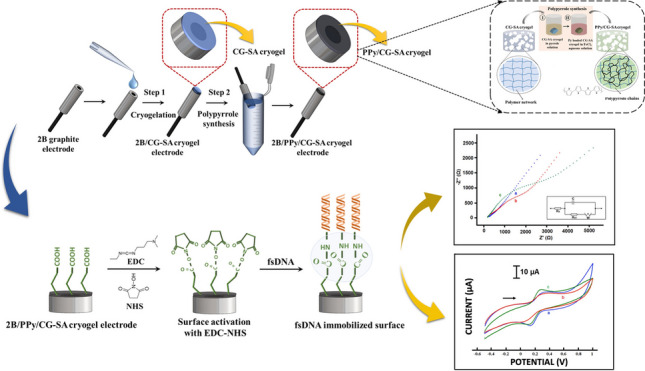

**Supplementary Information:**

The online version contains supplementary material available at 10.1007/s00604-024-06550-7.

## Introduction

Various studies have been conducted on human health for years, and studies are still ongoing. Information obtained as a result of these investigations has contributed to the development of new technologies used in the field of health. Technological developments, which continue to accelerate their development today, have led to innovations in biosensor technology. Biosensors, which date back to the 1960s, are devices that contain a combination of a biosensor element and a transducer [1]. Nucleic acids, enzymes, and antibodies are used as biosensing elements [2–4]. Biosensors are used in many different applications, such as the detection of disease-causing organisms in environmental applications, the assurance of food safety in the food industry, and the monitoring of the current state of agricultural products, as well as the detection/follow-up of diseases in medicine [5–7]. Electrochemical biosensors, which are among the types of biosensors classified according to transducer types, were found to be preferred over other types [8–10]. Electrochemical sensors come to the fore with their ease of use and cheapness. Various voltammetric techniques are used in electrochemical analysis on the basis of varying parameters and their combinations. Electrochemical systems can be analyzed by the cyclic voltammetry technique (CV) and electrochemical impedance spectroscopy (EIS). Cyclic voltammetry is a technique that allows the description of the kinetics of electron transfer reactions and reduction–oxidation processes. EIS is a surface-sensitive method that provides information about the charge transfer values of electrochemical reactions.

Studies are being carried out that offer new techniques to increase biosensor performance. Sensitivity, selectivity, and stability are the factors that determine the performance of a biosensor [11]. The sensitivity of biosensors can be improved by various methods. One of these methods is to design a functional intermediate material between the biomaterial and the artificial electrode [12]. The immobilization efficiency of biomolecules is an important factor in improving biosensor performance [13]. Thanks to functional intermediate materials, the immobilization capacity of biomolecules has improved. Cryogels are three-dimensional materials that are very suitable for intermediate materials with their spongy and macroporous (1–100 µm) structure [14–16]. These porous polymeric materials provide a large surface area on the electrode and increase its ability to hold biomolecules. Naturally derived polysaccharides are alternative materials for the preparation of cryogels. Macroporous cryogels are prepared with various polysaccharides [17–19]. Carrageenan (CG) and sodium alginate (SA) are biocompatible polysaccharides with gel formation capacity [20, 21]. Conducting polymers not only provide the connection between biomolecules and the electrode but also contribute to improving the electron transfer rate on the electrode surface. Polypyrrole (PPy) is a conducting polymer that is used for this purpose. It comes to the fore in biosensor studies due to its more stable and relatively higher electrical conductivity compared to other conductive polymers [22–25]. Conducting polymers can be used alone or synthesized in networks of polymeric materials, such as cryogels and hydrogels, for different purposes [26–28]. Conductive cryogels are obtained by the polymerization of pyrrole in cryogel networks [29–31].

Deoxyribonucleic acid (DNA) can be thought of as a database that carries genetic information. The biological importance of DNA is undisputed. Electrochemical DNA biosensors are an effective technology frequently used for the detection of inherited diseases, DNA sequence defects, infectious diseases, and cancer research [32–37].

Electrochemical biosensor studies based on biopolymers are available in the literature [24, 38–40]. For example, Liu et al. [38] developed an electrochemical biosensor based on glassy carbon electrode (GCE) modified with aldehyde-agarose hydrogel films for the detection of PML/RARa fusion gene for acute promyelocytic leukemia (APL). A chemical treatment with NaIO_4_ was carried out to convert diol groups in agarose gels to aldehyde groups. As a result of this process, agarose gels were converted into active hydrogel films containing aldehyde group [38]. A chronocoulometric sensor for miRNA-9–2 determination using a κ-carrageenan hydrogel-coated mesoporous gold (Au) electrode was reported. With κ-carrageenan gel on the gold electrode surface, target miR-9–2 determination was performed in the presence of a redox molecule-ruthenium hexaammine(III) chloride ([Ru(NH_3_)_6_]^3+^,RuHex) by performing “magnetic ısolation and purification” process. Then, this sample was incubated at electrode surface and measurument was done [39].

In the one of our earlier study [40], an electrochemical biosensor was developed for detection of drug-DNA interaction on the surface of homopolysaccharide biopolymer levan (LVN)–modified pencil graphite electrode. The interaction between the anticancer agent daunorubicin (DNR) and fsDNA was studied by differential pulse voltammetry technique by monitoring DNR and guanine oxidation signals. This study specifically reports results in the absence/presence of fsDNA interaction with DNR while reporting the LOD values for fsDNA (i.e., 2.74 μg mL^−1^) and for DNR (i.e., 510 nM) [40].

Electrochemical biosensor studies containing kappa-carrageenan and polypyrrole are also available in the literature [24]. Esmaeili et al. [24] developed a DNA biosensor based on kappa-carrageenan-polypyrrole-gold nanoparticle (KC-PPy-AuNPs) nanobiocomposite. Arowana fish DNA probe sequence extracted from male Arowana fish scales was immobilised to the DNA biosensor. The application of the DNA biosensor for sex classification of Arowana fish has been demonstrated [24].

This study presents an innovative modification material for development of electrochemical biosensor developed by a polysaccharide-based cryogel with a conductive polymer as an intermediate material and its application to DNA detection. The porous cryogels providing a large surface area on the graphite electrode were used to increase the amount of nucleic acids immobilized on the electrode surface.

The results of the synthesis and characterization studies of CG, CG-SA, PPy/CG, and PPy/CG-SA cryogels used as intermediate materials are reported. No report has been available in the literature yet on the use of carrageenan- and sodium alginate–based cryogels as an intermediate material for the development of biosensors.

Polypyrrole conductive polymer was chemically synthesized in a cryogel network and included in the structure to accelerate electron transfer. The developed PPy-cryogel-modified electrode was then applied for the determination of fsDNA. The experimental conditions for the modified electrode were optimized on the basis of electrochemical measurements using EIS and CV techniques. The linear range, detection limit with its sensitivity, and storage stability of the DNA biosensor were investigated.

## Materials and methods

### Apparatus and chemicals

All measurements were performed using a three-electrode system and also in a faraday cage by AUTOLAB PGSTAT-204 and AUTOLAB 302 (Eco Chemie, Utrecht, the Netherlands) with NOVA 1.11 software and the FRA module. For electrochemical measurements, CV and EIS were used. The three-electrode system consisted of a graphite electrode (Bhile, 2B) as the working electrode, a platinum wire as an auxiliary electrode, and Ag/AgCl/3 M KCl as the reference electrode (BAS, Model RE-5B, W. Lafayette, USA).

The cryogels of CG, CG-SA, PPy/CG, and PPy/CG-SA were characterized by the Fourier transform infrared spectrophotometer (FTIR) Bruker Tensor 27 FTIR (Bruker, USA) and the thermogravimetric analyzer (TGA) SII EXSTAR6000 TGA (Seiko Instruments Inc.).

Characterization of surface morphologies of modified electrodes (Bare 2B, 2B/PPy, 2B/CG cryogel, 2B/PPy/CG cryogel, 2B/CG-SA cryogel, and 2B/PPy/CG-SA cryogel) was performed using a HITACHI Regulus 8230 scanning electron microscope (SEM) and analyzed by energy-dispersive X-ray spectroscopy (EDX) (HITACHI, Tokyo, Japan).

Synthesis of CG, CG-SA cryogel in 3D/cross-linked form, and kappa (κ)-carrageenan (MW 788.647 g mol^−1^, Geel, Belgium) was purchased from Acros Organics; sodium alginate and sodium hydroxide (98%) were purchased from Sigma-Aldrich (Germany). Glycerol diglycidyl ether (GDE, MW 204.22 g mol^−1^) was purchased from Merck and used as a crosslinker. For the synthesis of PPy/CG, PPy/CG-SA, iron (III) chloride hexahydrate (FeCl_3_·6H_2_O, MW 270.33 g mol^−1^, Germany) was purchased from Merck, and pyrrole (98 + %) was purchased from Alfa Aesar. The polyurethane used to coat the 2B pencil electrode was obtained from Polikor.

1-Ethyl-3-(3-dimethylaminopropyl carboimide) hydrochloride (EDC) and N-hydroxysuccinimide (NHS) used as covalent chemical binders were purchased from Sigma-Aldrich.

Fish sperm DNA (fsDNA) was purchased from Sigma-Aldrich. Of fsDNA stock solution, 1000 µg mL^−1^ was prepared in ultra-pure water (Sigma) kept at − 20 °C, and it was diluted with acetate buffer solution (pH 4.80) (ABS). Double-distilled water (DW) was used in the experiments. All chemicals were used in analytical reagent grade.

### CG cryogel and CG-SA cryogel synthesis

Before the modified electrode was prepared, the electrode intermediate material cryogels were externally synthesized, and characterization studies were carried out. To determine the optimum synthesis parameters, the physical properties of the cryogels were observed by varying the solution concentration (Table S1), crosslinker ratio (Table S2), CG and SA solution mixing ratio (Table S3), and cryogelation time (Table S4). Optimum conditions were determined and recorded (Table S5). For the production of CG and CG-SA cryogels with the best properties, 0.05 g of CG was dissolved in 5 mL of 0.2 M NaOH at 50 °C for 2 h. For the sodium alginate solution prepared separately for CG-SA cryogels, SA weighing 0.05 g was dissolved in 5 mL of DW at room temperature. A CG-SA mixture (1:1, v/v) was prepared for CG-SA cryogel synthesis. The CG solution prepared for CG cryogel and the CG-SA mixture prepared for CG-SA cryogel were cooled in an ice bath under controlled conditions. The cooled solutions were vortexed by adding GDE used as a crosslinker at 20 mol% of the CG replicate unit. The mixture was then transferred to syringes. For cryogelation, it was kept at − 20 °C for 48 h. At the end of the period, the cryogels taken from the syringes were washed with DW three consecutive times for 20 min. When the washing process was complete, the cryogels were dried using the lyophilization technique.

### PPy/CG cryogel and PPy/CG-SA cryogel synthesis

PPy/CG and PPy/CG-SA conductive cryogels were externally synthesized and characterized to obtain detailed information about their structures. Conductive cryogels were synthesized with the guidance of the method available in the literature [41]. Pieces of CG and CG-SA cryogel were placed in a 0.5-M solution of pyrrole in 20 mL of DW. For polypyrrole polymerization, cryogels were loaded with pyrrole monomer for 30 min. At the end of the time, the cryogels were placed in a 0.5-M, 20-mL FeCl_3_ aqueous solution used as an oxidizing agent for the polymerization of pyrrole absorbed by the CG and CG-SA cryogels in the cryogel and kept for 2 h. Thus, the synthesis of PPy in cryogel networks was completed. After polymerization was completed, the PPy/CG cryogel and the PPy/CG-SA cryogel were washed three consecutive times with DW for 20 min. After being washed, the cryogels were dried in a freeze dryer.

### Characterization of CG, CG-SA, PPy/CG, and PPy/CG-SA cryogels

The SEM images of CG, CG-SA, PPy/CG, and PPy/CG-SA cryogels were captured by scanning electron microscopy (SEM, Hitachi Regulus 8230). The cryogels were placed onto carbon tape-attached aluminum SEM stubs at room temperature after being coated with gold to a few nanometers in a vacuum, and then, surface morphology was investigated. All cryogels were dried by using a freeze dryer before SEM analysis.

The FTIR spectra of CG, CG-SA, PPy/CG, and PPy/CG-SA cryogels were recorded using a FTIR spectrophotometer (TENSOR 27, Burker, Germany) with a range of 400–4000 cm^−1^ where 32 averaged scans were run with a resolution of 4 cm^−1^. The dried cryogels were ground and mixed with KBr to prepare disks. Then, the FTIR spectra of the cryogels were recorded using the OPUS program.

The thermal gravimetric analysis of CG, CG-SA, PPy/CG, and PPy/CG-SA cryogels was conducted (TGA, SII EXSTAR 6000, Japan). About 3–5 mg of cryogel was put into a ceramic pan, and the weight loss was recorded from 50 °C to 900 °C. The analysis was realized under an inert atmosphere using N_2_ (flow rate of 200 mL min^−1^) with a controlling temperature of 50–900 °C and a constant heating rate of 10 °C min^−1^.

### Electrode fabrication

The purchased 2B pencil tips with a diameter of 2.00 mm and a length of 120 mm were cut to dimensions of 4.0 cm. The cut 2B electrodes were coated with commercial polyurethane and allowed to dry for 2 days to obtain a stable surface, enabling cryogel synthesis. One centimeter of the dried polyurethane-coated pencil tips were cut straight. Two different types of sandpaper, 220 and 1200, were used to smooth and clean the electrode surfaces. In the last step, the surfaces of the electrodes and coated parts were cleaned with DW and ethanol.

### Preparation of 2B/PPy/CG cryogel and 2B/PPy/CG-SA cryogel-modified electrodes

A cryogel solution of CG and CG-SA (1:1, v/v) (10.0 mg mL^−1^) was prepared. A volume of 3 µL of the prepared solution was dripped onto the cleaned electrode surfaces. After completion of the cryogelation step, the electrodes were dipped into vials containing 0.5 L, 0.5 M pyrrole solution in groups of three for the chemical synthesis of polypyrrole onto the electrode surface. The reaction continued for 30 min. The electrodes were kept in an oxidizing 0.5 L, 0.5 M FeCl_3_ aqueous solution for 2 h to complete polymerization after 30 min. Then, it was washed with DW three consecutive times for 10 s (see the “Modified electrode characterization and optimization” section).

### Covalent surface activation of the modified electrode

For covalent activation of the 2B/PPy/CG cryogel and the 2B/PPy/CG-SA cryogel-modified electrodes, 5 µL of a solution of 4 mM EDC (1-ethyl-3-(3-dimethylaminopropyl carboimide) hydrochloride) and 1 mM NHS (N-hydroxysuccinimide) [42] covalent agent prepared in phosphate buffer solution was dropped onto the electrode surfaces. The activation was carried out at room temperature for 15 min.

### Immobilization of fsDNA on 2B/PPy/CG cryogel and 2B/PPy/CG-SA cryogel surfaces

For immobilization of fsDNA, 5 µL of fsDNA solution prepared in acetate buffer solution at different concentrations was dropped onto the surfaces of modified electrodes 2B. The electrodes were left at room temperature for 30 min to complete immobilization.

Therefore, the difference in the electrochemical behavior of cryogel electrodes 2B/PPy /CG-SA containing carboxyl groups (-COOH) and cryogel electrodes 2B/PPy/CG without -COOH groups after activation of EDC-NHS was observed [35, 43–46].

### Optimization of modified electrode composition

For the modified electrode composition, the concentration of the cryogel solution (15.0, 10.0, 5.0 mg mL^−1^) volume of solution dripped onto the electrode surface (1 µL, 2 µL, 3 µL, 4 µL), the pyrrole monomer concentration (0.25 M, 0.5 M, 0.75 M), and the polypyrrole synthesis reaction time (15 min, 30 min, 45 min, and 60 min) were optimized.

### Electrochemical measurements

The voltammetric measurements were performed using the cyclic voltammetry (CV) technique in 2.5 mM redox probe containing K_3_[Fe(CN)_6_]/K_4_[Fe(CN)_6_] (1:1) and 0.1 M KCl applying a step potential of 25 mV, a scan rate of 50 mV s^−1^; forward scan, − 0.5 to + 1 V; and reverse scan, + 1 V to − 0.5 V. Measurements were made on the redox probe signal at + 0.2 V.

Impedimetric measurements were performed in the presence of 2.5 mM Fe(CN)_6_^3−/4−^ prepared in 0.1 M KCl. The impedance was measured in the frequency range between 100 mHz and 100 kHz at a potential of + 0.23 V with a sinusoidal signal of 10 mV. The frequency interval is divided into 98 logarithmically equidistant measure points. The elements of Randles circuit values were calculated using the fitting programme of NOVA (version 1.11, EcoChemie, the Netherlands). The equivalent circuit model (Randles circuit) is used to fit the data obtained by impedimetric measurements, which consist of charge transfer resistance (*R*_*ct*_), solution resistance (*Rs*), Warburg impedance (*W*), and constant phase element (*C*). The respective semicircle diameter corresponds to *R*_*ct*_. The related circuit model is used to fit the impedance data, as shown in all figures containing Nyquist diagrams that are plotted by using the data obtained by EIS measurements. All data presented in each Nyquist diagram is the fitted version of the related data.

## Results and discussion

### Cryogel synthesis and characterization

**Fig. 1:**
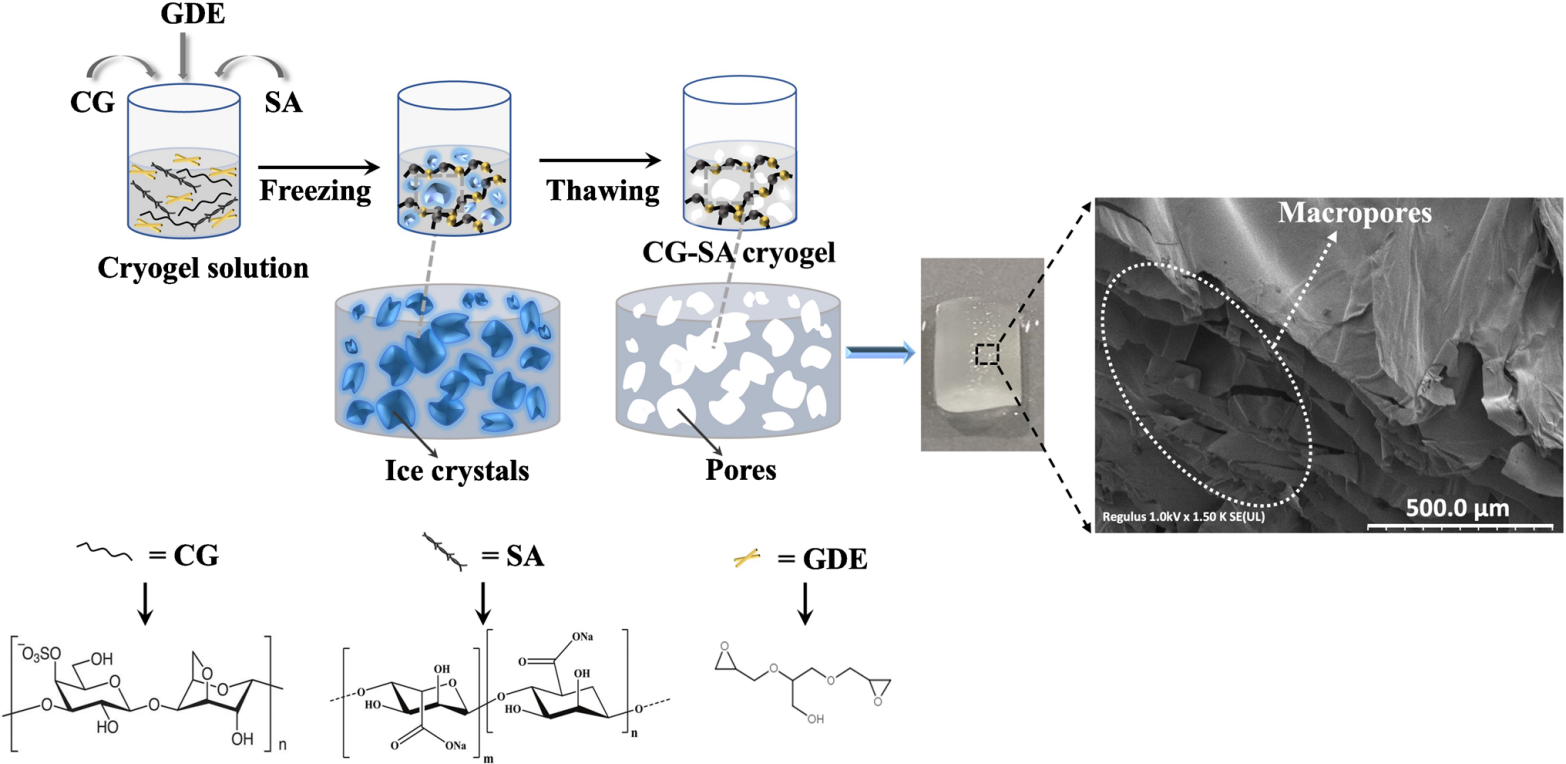
Schematic representation of CG-SA cryogel synthesis and SEM image of the cryogel (scale bar 500 µm)

To better understand the structures of cryogels, we can give an example of the structures of sponges that are frequently encountered in daily life. When the sponge is mentioned, the first features that come to mind are their high-water absorption capacities and their porous structures. Cryogels are also spongy gels with macropores. All stages of the formation of this spongy and porous structure are called the cryogelation process [47]. In other words, the cryogelation process refers to the formation of porous gels at sub-zero temperatures. For the synthesis of CG and CG-SA cryogels, a mixture of CG and CG-SA was dissolved in a NaOH solution. The mixture was cooled in a controlled manner. GDE was added to the cooled reaction mixture and incubated at −20 °C for 48 h to complete cryopolymerization. After cryogel formation, the CG and CG-SA cryogels were cleaned with DW for about 3 h, changing DW frequently. Fig. 1 shows a schematic representation of the synthesis of cryogels of CG and CG-SA (Fig. 1). The results of the optimization study of cryogels synthesized at different synthesis parameters are presented in Supplementary Materials.

PPy is highly preferred in biomedical applications because it is environmentally stable, easy to synthesize, and can maintain its electrical properties and biocompatibility during the experiment. There are different preparation techniques such as chemical polymerization and electrochemical polymerization. In addition to the advantage of PPy, its fragility is a disadvantage and this causes limitations especially in biosensor applications. It is used in combination with different natural and synthetic polymers to eliminate this disadvantage and to prepare/synthesize a mechanically strong conductive composite [24, 25]. When PPy is added to carrageenan, the composite polymer precipitates on the solid surface, changing from hydrophilic to hydrophobic, while maintaining its water permeability. The structure, morphology, and conductivity of the carrageenan-PPy nanocomposite have been attributed to its success in producing electrically conductive composites offering a wide range of interesting mechanical and electrical properties for the fabrication of DNA biosensor [48].

In this study, the electrical conductivity of PPy was utilized, but PPy was not used directly. Firstly, carrageenan (carrageenan-alginate), a natural polymer, was chemically prepared as a 3D cryogel form by using epoxy based cross-linker. This prepared macroporous 3D structure was used as a template, and pyrrole was adsorbed to these networks, and then, pyrrole was polymerised. In addition, the cryogel support material can be modified and the new functional groups can be added into the structure. Besides these advantages, the structure can be designed analyte-specific for the analysis of different materials. Also, less material (monomer/polymer) is used in cryopolymerization. One of the advantageous aspects of our study is that the polysaccharides used in cryogel synthesis are cheap, biocompatible, and easily accessible.

**Fig. 2:**
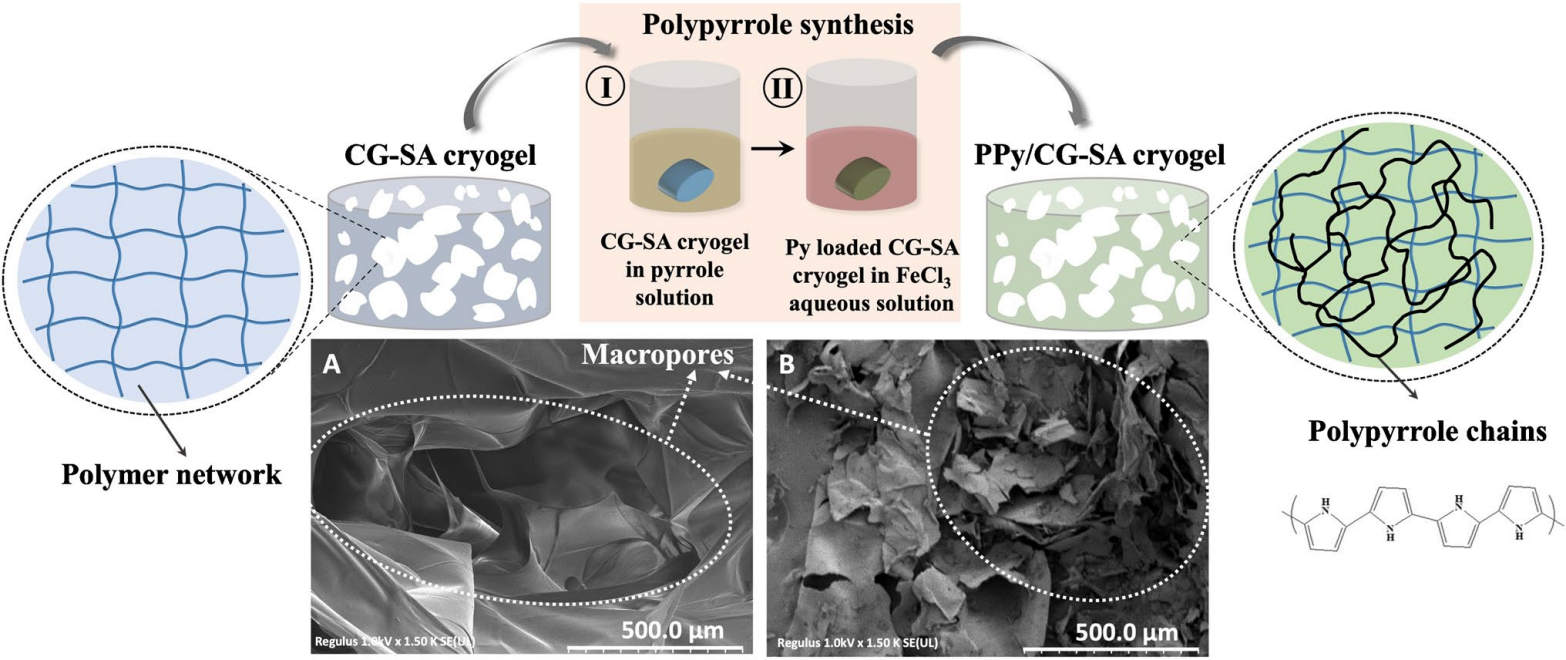
Schematic representation of PPy/CG-SA cryogel synthesis by using CG-SA cryogel as template and SEM images of the cryogels: (**A**) CG-SA cryogel, (**B**) PPy/CG-SA cryogel (scale bar 500 µm)

Polypyrrole synthesis was performed inside the CG and CG-SA cryogels to obtain conductive cryogels. Cryogels were taken into an aqueous pyrrole solution and kept in this solution for 30 min. Then, it was kept in a FeCl_3_ aqueous solution for 2 h to complete polymerization. A schematic representation of the synthesis of PPy/CG-SA cryogels and SEM images of CG-SA and PPy/CG-SA cryogels is presented in Fig. 2.

Although the porous structures of the CG-SA cryogels can be seen in SEM images, the pores cannot be clearly seen in the SEM image after PPy synthesis. This is because PPy fills the pores of CG-SA cryogels.

To obtain more detailed information about the structures of cryogels synthesized for electrode surface modification, the chemical structures of CG cryogel, CG-SA cryogel, PPy/CG cryogel, and PPy/CG-SA cryogel were determined by FTIR analysis. Thermogravimetric analyses of cryogels were performed in the temperature range of 30–1000 °C to determine their thermal stability.

The FTIR spectra of CG cryogel and PPy/CG cryogel are presented in Fig. S1A, and the FTIR spectra of CG-SA cryogel and PPy/CG-SA cryogel are presented in Fig. S1B. In the spectrum given for CG cryogels, the large peaks present at wave numbers of 3429–3441 cm^−1^ for both the conductive and nonconductive groups are due to the hydroxyl (-OH) group in the structure of the cryogels, and the peaks at 2923–2924 cm^−1^ are due to CH stretching. The characteristic peaks of CG cryogels are given by the peaks at 1257 cm^−1^, 1070 cm^−1^ and 846 cm^−1^. Sulfate stretch of 1257 cm^−1^, glycosidic bonds (C–O–C) at 1070 cm^−1^, and absorption at 846 cm^−1^ are attributed to galactose-4-sulfate (C-O-SO_3_) [49, 50].

In the PPy/CG cryogel spectrum, the absorption C = C stretch band observed at 1547 cm^−1^ corresponds to the fundamental vibration of the polypyrrole ring [51, 52]. This absorption band determines the presence of PPy synthesis. The peak at 1163 cm^−1^ is due to the C-N stretching vibration.

In the CG cryogel spectrum, after PPy synthesis, it was observed that the peak at 1257 cm^−1^ shifted to the peak at 1288 cm^−1^, and the peak at 1070 cm^−1^ shifted to the peak at 1041 cm^−1^.

-OH peaks were observed at 3401 cm^−1^ and 3425 cm^−1^ in the FTIR spectra of cryogels containing SA given in Fig. S1B. Peaks at 2908–2925 cm^−1^ were attributed to C-H stretching vibration. Asymmetric stretching of -COO- groups at 1620 cm^−1^ and -COO- symmetrical stretching vibration at 1419 cm^−1^ give the characteristic peaks of SA [53]. Peaks at 1257 cm^−1^, 1042 cm^−1^, and 846 cm^−1^ were attributed to sulfate stretching (-SO_3_), glycosidic bonds (C–O–C), and galactose-4-sulfate (C-O-SO_3_) stretching, respectively, which are characteristic of carrageenan. The characteristic peaks of CG and SA confirm the synthesis of cross-linked CG-SA cryogels. In addition to the characteristic peaks of CG and SA in the FTIR spectrum of PPy/CG-SA cryogel, PPy ring vibrations, the C = C band at 1544 cm^−1^ peak and C-N stretching peak seen at 1164 cm^−1^ confirm PPy synthesis.

Details of the results of the TGA analysis are presented in the Supporting Information. The TG curves of CG and PPy/CG cryogels are given in Fig. S2.

The TGA results showed that the polypyrrole carrageenan-sodium alginate cryogels were more thermally stable than the carrageenan-sodium alginate cryogels (Fig. S2).

### Modification of the working electrode

**Fig. 3:**
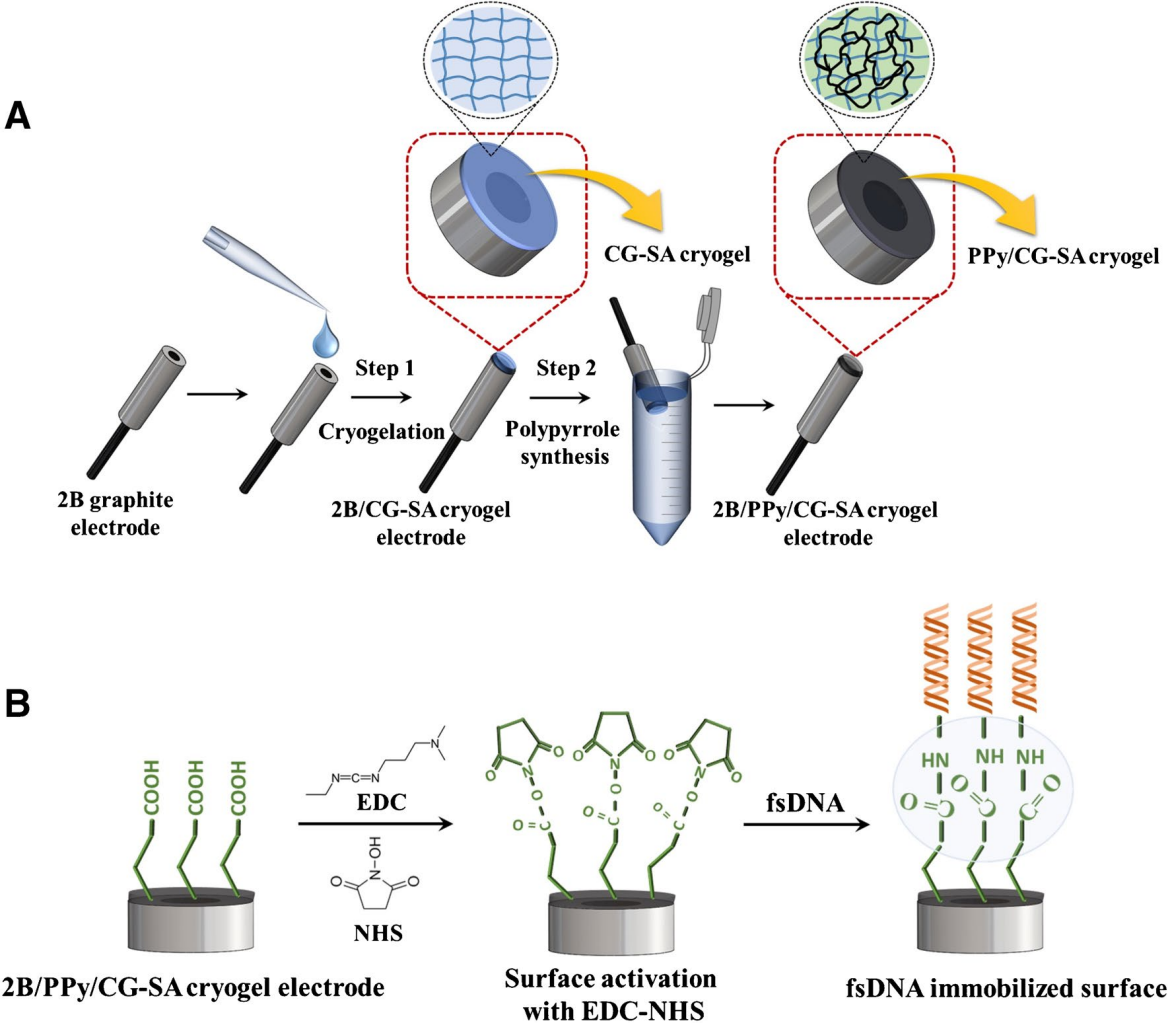
**A** Schematic representation of the electrode modification. **B** Schematic showing the immobilization of fsDNA on the electrode surface activated by EDC-NHS

In other biosensor studies using cryogel as an intermediate material, working electrodes such as gold electrodes [54–56] or screen-printed electrodes (SPE) [57, 58] were generally used. In this study, insulator-coated 2B pencil tip electrode surfaces were modified with CG and CG-SA cryogel. In the first stage, cryogel synthesis was completed, and in the second stage, chemical synthesis of polypyrrole was carried out on the electrode surface (Fig. 3A).

After the modification step was completed, the 2B/PPy/CG cryogel and 2B/PPy/CG-SA cryogel–modified electrode surfaces were activated with the EDC-NHS covalent coupling agent as shown in Fig. 3B. The determination of fsDNA was performed with electrodes with activated surfaces.

In the group containing sodium alginate, it was concluded that the surface became more stable thanks to the -COOH groups in the sodium alginate structure and the measured signals were more reproducible. At the same time, it allows modification due to the fact that it contains -OH and -COOH functional groups in the structure and makes it possible for new groups to transform into ester, amide, ether, and other structures with different bonds.

### Modified electrode characterization and optimization

**Fig. 4:**
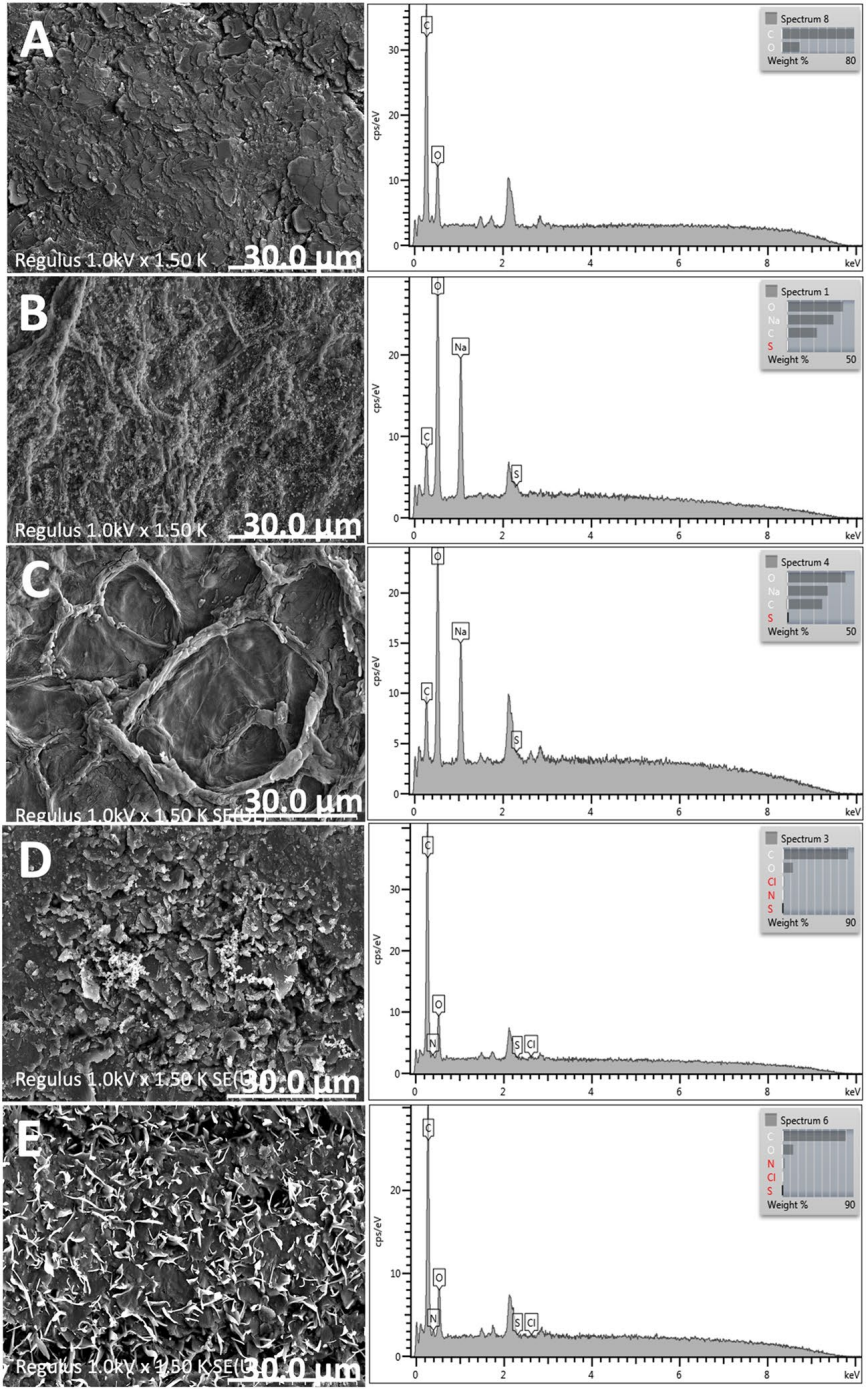
The EDX spectrum of 2B electrode (**A**), 2B/CG cryogel (**B**), 2B/CG-SA cryogel (**C**), 2B/PPy/CG cryogel (**D**), and 2B/PPy/CG-SA (**E**) (scale bar 30 µm)

SEM energy-dispersive X-ray spectroscopy (EDX) analysis was performed for the surface characterization of modified electrodes (Fig. 4). For the SEM analysis, air-dried 2B electrode, 2B/CG cryogel electrode, 2B/CG-SA cryogel electrode, 2B/PPy/CG cryogel electrode, and 2B/PPy/CG-SA cryogel electrode werre cut into upper side about 3–5 mm. The cutted cryogel electrodes were placed onto carbon tape-attached aluminum SEM stubs at room temperature after coating with gold to a few nanometer thickness in a vacuum, and then, surface morphology was investigated. According to the results, as expected, the presence of C and O elements in the bare 2B electrode and the presence of S and Na elements on the surface after the modifications of the CG cryogel and the CG-SA cryogel was confirmed. After the PPy/CG cryogel and PPy/CG-SA cryogel modifications, the N element originating from the pyrrole ring shows that PPy synthesis has taken place on the electrode surface.

Details of the optimization work for the modified electrode composition are reported in the Supplementary Materials. The conditions determined for surface modification are as follows: The volume of solution dropped on the electrode surface was 3 µL (Table S6 and Fig. S3), and the change in the average Ia values of the electrodes prepared at different solution volumes was investigated (Table S7). The change in the average Ia values of electrodes prepared at pyrrole monomer concentration of 0.5 M (Table S8 and Fig. S4), polypyrrole synthesis time of 30 min (Table S9 and Fig. S5), and different polypyrrole synthesis times is presented in Table S10. CG cryogel solution of 10.0 mg mL^−1^ (Table S11 and Fig. S6) and the change in the average Ia values of the electrodes due to the difference in the concentration of the cryogel solution were calculated (Table S12). Conditions for optimum electrode composition are summarized in Table S13.

The electrodes prepared under the determined optimum conditions were characterized using the cyclic voltammetry technique. The electrochemical characterization of the modified electrodes was performed in a 0.1-M KCl solution and a redox solution containing 2.5 mM Fe (CN)_6_^3−/4−^ (Fig. S7).

The surface areas calculated for the 2B electrode and the modified electrodes were calculated according to the Randles–Sevcik equation [59] that is given in Eq. 1:

1 $$Ip=\left(2.69\times {10}^{5}\right) {n}^\frac{3}{2} A\;C\;{D}\;^\frac{1}{2} {V}^\frac{1}{2}$$

where $Ip$ is the peak current, $n$ is the number of electron transferred, $\text{A}$ is the electroactive surface area (cm^2^), $C$ is the concentration (mol cm^−3^), $D$ is the diffusion coefficient (7.6 × 10^−6^ cm^2^ s^−1^), and $V$ is the scan rate (V s^−1^).

According to the calculated values, the surface area for the 2B electrode was recorded as 0.016 cm^2^. Volumes of cryogel solution (1 µL, 2 µL, 3 µL, and 4 µL) were dripped, and the surface area was calculated for the prepared electrodes. Compared to the 2B electrode, an increase in the surface area of the modified 2B/PPy/CG cryogel electrodes was recorded using solution in volumes of 1 µL (*A* = 0.035 cm^2^), 2 µL (*A* = 0.046 cm^2^), 3 µL (*A* = 0.045 cm^2^), and 4 µL (*A* = 0.034 cm^2^) (Table S6).

The surface areas of the electrodes prepared at different polypyrrole synthesis times (15, 30, 45, and 60 min) were evaluated. The effective surface area of each of the electrodes (2B, 15 min of 2B/PPy/CG cryogel, 30 min of 2B/PPy/CG cryogel, 45 min of 2B/PPy/CG cryogel, 60 min of 2B/PPy/CG cryogel) was calculated according to the Randles–Sevcik equation.

The surface area values of the modified electrodes were observed to increase with changing concentration of pyrrole monomers compared to the 2B electrode (Table S8).

When the surface areas of 2B/PPy/CG cryogel electrodes modified at different PPy synthesis times were evaluated, it was determined that the surface area value of 0.016 cm^2^ for the 2B electrode increased by 1.81 times for 15 min 2B/PPy/CG cryogel electrode, by 2.94-fold for 30 min 2B/PPy/CG cryogel electrode, by 3.06-fold for 45 min 2B/PPy/CG cryogel electrode, and by 2.94 times for 60 min 2B/PPy/CG cryogel electrode (Table S9).

In addition, similar to the other results, the concentration optimization studies of the cryogel solution also showed an increase in the surface area values compared to the 2B electrode (Table S11).

The results showed that the synthesized polymers on the electrode surface have the effect of increasing the surface area depending on the structure of both macroporous cryogels and PPy.

### The electrochemical optimization of volume of solution dripped onto the electrode surface

The effect of the volume of CG cryogel synthesized on the electrode surface on the electrode response was investigated. The cryogel was synthesized by dropping 1 μL, 2 μL, 3 μL, and 4 μL volumes of cryogel solution at a concentration of 10.0 mg mL^−1^ onto the electrode surface, and then, pyrrole was loaded into CG cryogels in 0.5 M, 0.5 mL aqueous pyrrole solution for 30 min. Then, CV measurements of the modified electrodes were performed. The results obtained are shown in Fig. S3. Average anodic peak current (Ia) and cathodic peak current (Ic), anodic relative charge (Qa) and cathodic relative charge (Qc), and calculated electroactive surface area (A) data were obtained for electrodes with different volumes of cryogel solution on their surfaces as shown in Table S6. The average values of Ia increased for all modified electrodes (Table S7).

The highest increase was observed at electrodes with cryogel volumes of 2 μL (65.0%) and 3 μL (64.0%). It was observed that the increase in the anodic peak current values of the 2B/PPy/CG cryogel–modified electrodes with polymeric film volumes of 2 μL and 3 μL was close to each other. Since the signals measured at 2B/PPy/CG cryogel electrodes with cryogel solution volume of 3 μL were more reproducible (RSD %, 3.30%,* n* = 2), the optimum volume of cryogel solution dripped onto the surface was determined as 3 μL.

### The electrochemical optimization of PPy monomer concentration

For the pyrrole concentration optimization study, the concentration of the CG cryogel solution on the 2B electrode surfaces was kept constant at 10.0 mg mL^−1^, the pyrrole loading time was 30 min, and the solution volume was 3 μL. Polypyrrole conductive polymer was synthesized on the 2B/CG cryogel electrode with pyrrole solutions prepared as 0.25 M, 0.5 M, and 0.75 M. CV measurements were performed with electrodes prepared at different concentrations of pyrrole monomer (Fig. S4). For electrodes with different concentrations of pyrrole, the average anodic peak current (Ia) and cathodic peak current (Ic), the relative anodic charge (Qa), the relative cathodic charge (Qc), and the electroactive surface area (A) data are presented in Table S8.

The increase in anodic peak current values at modified electrodes prepared with 0.25, 0.5, and 0.75 M pyrrole solution was calculated as 53.9%, 65.4%, and 62.1%, respectively. The highest increase was seen in the electrodes with a concentration of 0.5 M pyrrole solution. Therefore, the optimum concentration of pyrrole solution was determined as 0.5 M.

### The electrochemical optimization of PPy synthesis reaction time

Ten milligrams per liter of cryogel solution concentration, 3 μL of cryogel solution volume, and 0.5 M of pyrrole monomer concentration remained constant, and the effect of PPy synthesis time was investigated. Polypyrrole synthesis was carried out for 15, 30, 45, and 60 min on the electrodes whose cryogelation process was completed. The behavior of the modified electrodes prepared at different synthesis times was investigated by the cyclic voltammetry technique (Figure S5). Electrodes prepared at different PPy synthesis times, average anodic peak current (Ia) and cathodic peak current (Ic), anodic relative charge (Qa), cathodic relative charge (Qc), and electroactive surface area (A) data are given in Table S9.

When the CV measurement results of the modified electrodes were compared with the 2B electrode, an increase in the average Ia values of all was observed (Table S10).

### The electrochemical optimization of cryogel solution concentration

Three microliters of CG solutions prepared at concentrations of 15.0, 10.0, and 5.0 mg mL^−1^ was dropped onto the surface of the 2B electrode. CV measurements of the modified electrodes were kept at − 20 °C for 48 h for cryogel synthesis, and then, PPy synthesis was performed in 0.5 M, 0.5 L aqueous pyrrole solution for 30 min. The results obtained are shown in Figure S6. Among electrodes prepared at different concentrations of CG solution, the average values of anodic peak current (Ia) and cathodic peak current (Ic), anodic relative charge (Qa), cathodic relative charge (Qc), and electroactive surface area (A) are shown in Table S11. When these data were evaluated, no peak current was measured in the redox probe solution by the electrodes modified with CG cryogel alone. This response was explained by the non-conductive nature of CG cryogels reducing electron transfer. Compared to the 2B electrode and the 2B/CG cryogel–modified electrode, an increase in the anodic currents of the modified electrodes was observed after the 2B/PPy/CG cryogel modification (Table S12). This result showed that PPy incorporated into the electrodes after 2B/PPy/CG cryogel modification significantly improved the conductivity and the PPy conductive polymer was synthesized inside the CG cryogels.

### Electrochemical behaviour of modified electrodes

**Fig. 5:**
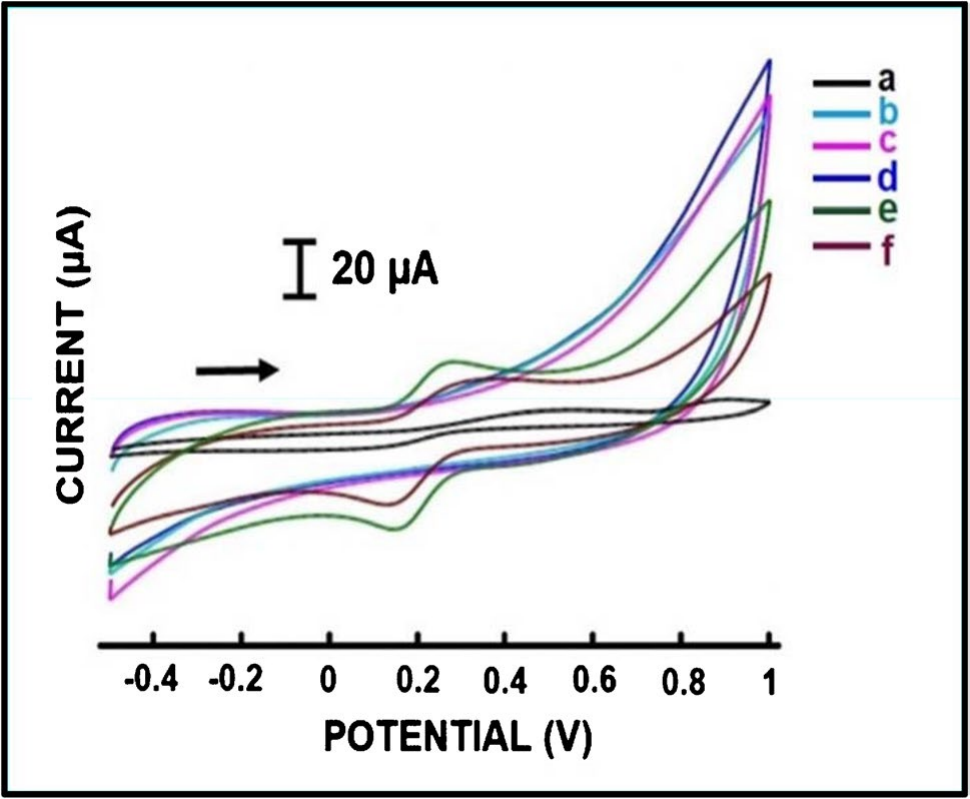
Cyclic voltammograms of 2.5 mM redox probe solution at (a) 2B, (b) 2B/SA, (c) 2B/CG cryogel, (d) 2B/CG/SA cryogel, (e) 2B/PPy/CG cryogel, and (f) 2B/PPy/CG-SA cryogel electrodes

After the optimization study, the behaviours of the 2B electrode, 2B/SA, 2B/CG cryogel, 2B/CG/SA cryogel, 2B/PPy/CG cryogel, and 2B/PPy/CG-SA cryogel electrodes were compared with the CV technique (Fig. 5; Fig. S7). The average values of the anodic peak current (Ia) of the results are summarized in Table S14.

According to the results summarized in the Table S14, no signal was observed for the 2B/SA, 2B/CG cryogel, and 2B/CG/SA cryogel–modified electrodes. The results were evaluated as SA, CG, and CG/SA cryogels synthesized on the electrode surfaces block electron transfer considering the insulating nature of both CG and SA. After the modification of the 2B/CG cryogel electrode with polypyrrole, the Ia value was 12.64 ± 0.66 µA, and after the modification of the 2B/CG-SA cryogel with polypyrrole, the Ia value was measured as 10.26 ± 1.27 µA. As expected, it was observed that electron transfer was accelerated thanks to the conductivity provided after the synthesis of polypyrrole on the electrode surface blocked with the cryogel.

Calculated surface area values (A) based on oxidation values for all modified electrodes are presented in Table S14. The calculated surface area for the 2B/PPy/CG-SA cryogel electrode is 0.030 cm^2^.

### Effect of EDC-NHS activation on 2B/PPy/CG cryogel and 2B/PPy/CG-SA cryogel electrodes

**Fig. 6:**
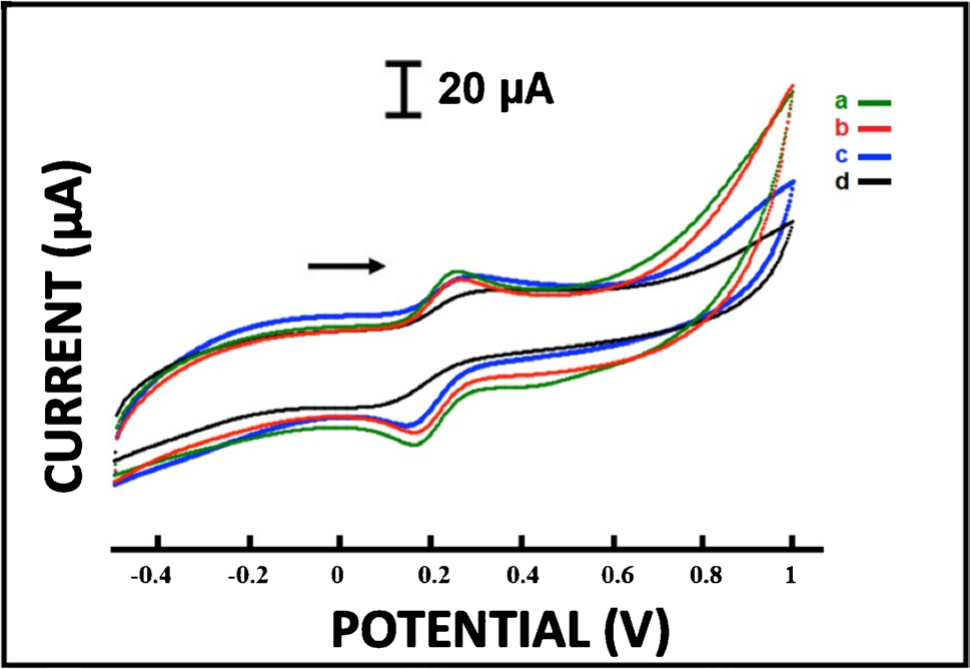
Cyclic voltammograms of 2.5 mM redox probe solution at (a) 2B/PPy/CG cryogel, (b) 2B/PPy/CG-SA cryogel, (c) 2B/PPy/CG cryogel/EDC-NHS, and (d) 2B/PPy/CG-SA cryogel/EDC-NHS

Electrochemical changes that occurred after the interaction of 2B/PPy/CG cryogel and 2B/PPy/CG-SA cryogel-modified electrodes with EDC-NHS were investigated (Fig. 6). The responses of the modified electrodes in the presence and absence of SA and 30 min of EDC-NHS interaction were investigated using the CV technique. The average values of the anodic peak current (Ia) and the relative standard deviation (RSD%) after activation of EDC-NHS and immobilization of fsDNA are listed in Table S15.

On the surface of the cryogel film containing sodium alginate polysaccharide, -COOH groups are present. When fsDNA is added to the EDC/NHS-activated surface, a covalent bond is formed between the amine group (-NH_2_) and the -COOH group on the guanine base of fsDNA. In DNA immobilization using reagents such as EDC/NHS, carboxyl groups on the surface are activated with the help of EDC/NHS reagents and then DNA molecules bind to these activated groups via amine groups. Thus, DNA is more stably bound to the electrode surface.

As seen in Table S15, the response to fsDNA was better in modified electrodes containing SA compared to electrodes without SA. The average anodic peak current value (Ia) of 2B/PPy/CG cryogel electrodes was found as 12.64 ± 0.66 µA (RSD %, 5.25%, *n* = 2) after interaction with EDC-NHS 7.51 ± 5.66 µA (RSD %, 75.35%, *n* = 2) and after fsDNA immobilization 9.47 ± 2.03 µA (RSD %, 21.48%, *n* = 2). At 2B/PPy/CG-SA cryogel, the average anodic peak current value (Ia) was calculated as 10.26 ± 1.27 µA (RSD %, 12.43%, *n* = 2), after the EDC-NHS interaction 10.14 ± 1.05 µA (RSD %, 8.39%, *n* = 2), and after fsDNA immobilization 14.10 ± 0.89 µA (RSD %, 6.30%, *n* = 2) (Table S15). When 2B/PPy/CG cryogel electrodes were compared with 2B/PPy/CG-SA cryogel/fsDNA electrodes, a 7.70% decrease in the average Ia values was observed in the final case, while an increase of 39.05% was recorded in 2B/PPy/CG-SA cryogel/EDC-NHS/fsDNA electrodes compared to 2B/PPy/CG cryogel-EDC-NHS electrodes.

When the results were examined, it was determined that more reproducible results were obtained with 2B/PPy/CG-SA cryogel/EDC-NHS/fsDNA electrodes. This was associated with a more stable electrode surface as a result of the covalent bonding of carboxylic groups (-COOH) in the structure of SA in 2B/PPy/CG-SA cryogel–modified electrodes with EDC-NHS. The improvement of RSD % as 6.30% after fsDNA immobilization on 2B/PPy/CG-SA cryogel/EDC-NHS electrodes confirmed this situation. Therefore, fsDNA determination was performed with electrodes containing SA.

### Effect of EDC-NHS interaction time at 2B/PPy/CG-SA cryogel–modified electrodes

The effect of EDC-NHS interaction time at 2B/PPy/CG-SA cryogel–modified electrodes was investigated using the CV technique (Fig. S8). After 15 and 30 min of EDC-NHS interactions, 50 µg mL^−1^ fsDNA immobilization was performed for 30 min. After 15 min of interaction with EDC-NHS and immobilization of fsDNA, the average anodic peak current (Ia) was measured as 9.52 ± 0.50 µA (RSD %, 5.28%, *n* = 2). In the case of the 30-min EDC-NHS interaction, the Ia value was recorded as 14.10 ± 0.89 µA (RSD %, 6.30%, *n* = 2). When the results were evaluated, it was seen that the RSD % value improved as 5.28% in the case of 15 min of EDC-NHS interaction. According to these results, the optimal interaction time of EDC-NHS was determined to be 15 min.

### CV and EIS characterization of 2B/PPy/CG-SA cryogel-modified electrodes

By performing fsDNA immobilization on electrode surfaces modified with 2B/PPy/CG-SA cryogel before and after EDC-NHS activation, the electrode surface was characterized using CV and EIS techniques. The results of the CV characterization study are presented in Table S16.

As a result of CV measurements (Fig. S9), the average anodic peak current (Ia) was obtained as 10.26 ± 1.27 µA and 5.89 ± 0.60 µA for 2B/PPy/CG-SA cryogel and 2B/PPy/CG-SA cryogel/EDC-NHS electrodes, respectively, whereas the average anodic peak current (Ia) was calculated as 7.63 ± 1.77 µA after the immobilization of 50 µg mL^−1^ fsDNA. For 2B/PPy/CG-SA cryogel electrodes, there was a 42.59% decrease in the anodic peak current (Ia) after EDC-NHS interaction and a 29.54% increase after 50 µg mL^−1^ fsDNA immobilization. The decrease in signal after EDC-NHS modification compared to 2B/PPy/CG-SA cryogel electrode was explained by the insulating effect of EDC-NHS molecules [60, 61].

**Fig. 7:**
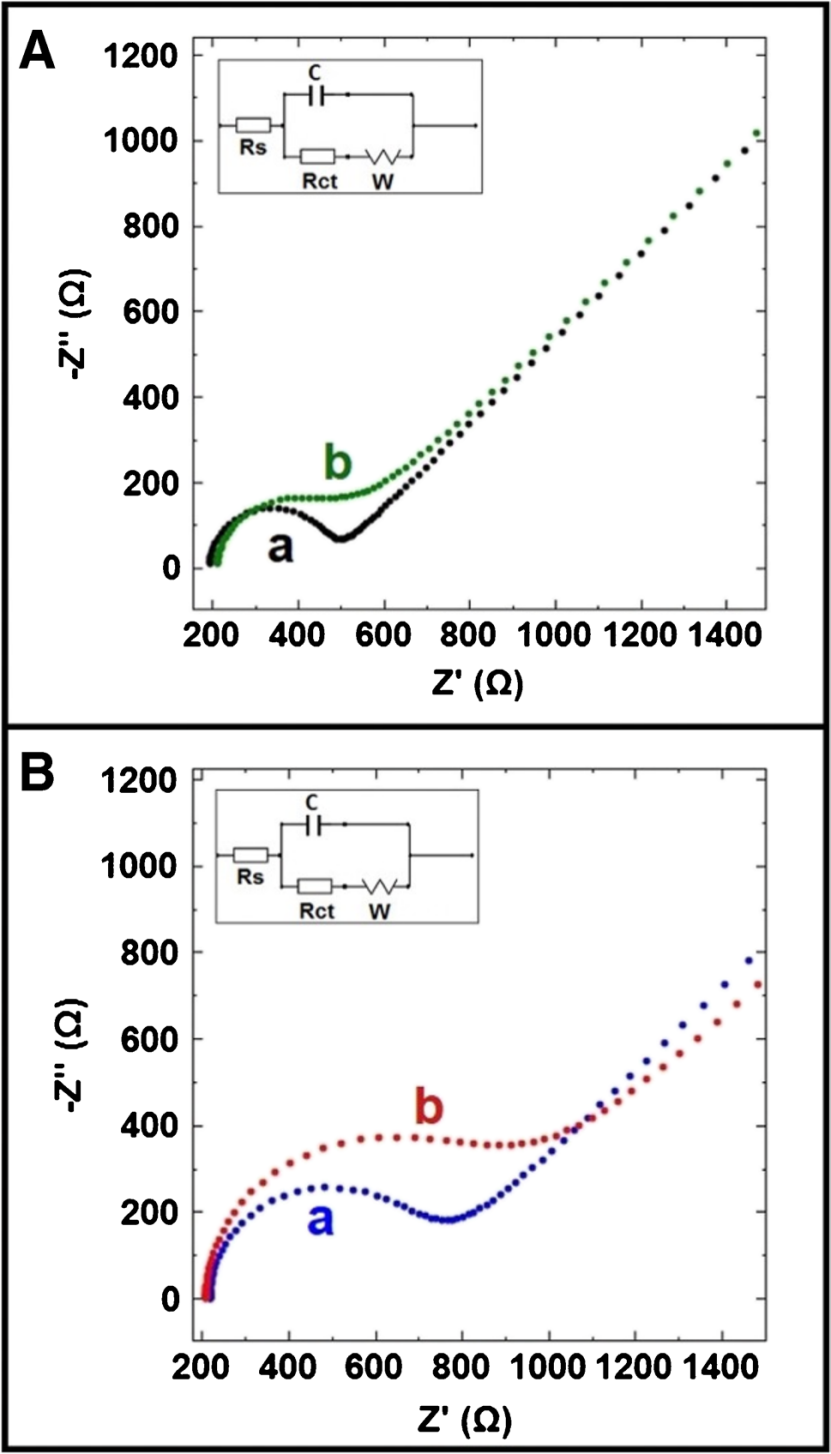
Nyquist curves obtained from (a) 2B/PPy/CG-SA cryogel and (b) after immobilization of 10 µg mL^−1^ fsDNA on the surface of (A) without EDC-NHS activation and (B) with EDC-NHS activation in 2.5 mM redox probe solution

In Fig. 7, the Nyquist diagrams were represented by EIS measurements. The characterization of the developed cryogel polysaccharide–based electrodes and the effect of EDC-NHS activation were investigated with the average charge transfer resistance (*R*_*ct*_) value (Table S17).

The change in *R*_*ct*_ after EDC-NHS activation and fsDNA immobilization was calculated according to Eq. 2:

2 $$\text{The change in the charge transfer resistance }= \frac{{R}_{ct1}}{{R}_{ct2}}$$

For the first conditions, the charge transfer resistance after EDC-NHS activation is expressed as *R*_*ct*1_ and the charge transfer resistance before EDC-NHS activation is expressed as *R*_*ct*2_. For the second conditions, the charge transfer resistance after fsDNA immobilization is expressed as *R*_*ct*1_ and the charge transfer resistance before fsDNA immobilization is expressed as *R*_*ct*2_.

While the average *R*_*ct*_ value obtained with 2B/PPy/CG-SA cryogel–modified electrodes was as 201 ± 101 Ω (RSD % = 50.43%, *n* = 2), the average *R*_*ct*_ value obtained after EDC-NHS activation was measured as 396 ± 110 Ω (RSD % = 27.86%, *n* = 2). The change on the surface of the 2B/PPy/CG-SA cryogel–modified electrodes after EDC-NHS activation was observed as a 97.51% increase. According to the results obtained, a stable electrode surface was obtained after EDC-NHS activation and more repeatable results were obtained (Table S17).

After 10 µg mL^−1^ fsDNA immobilization, 45.58% increase was obtained EDC-NHS activated 2B/PPy/CG-SA cryogel–modified electrodes in contrast to 2B/PPy/CG-SA cryogel–modified electrodes as shown as Fig. 7, and also, more repeatable results (577 ± 66 Ω, RSD % = 11.41%, *n* = 2) were obtained.

This increase at the value of *R*_*ct*_ after fsDNA immobilization indicated a decrease in electron transfer resistance and a change at the electrode surface. Changes after EDC-NHS activation and fsDNA immobilization can be evaluated as fsDNA is immobilized on the electrode surface. The apparent fractional coverage values (${{\varvec{\theta}}}_{{\varvec{I}}{\varvec{S}}}^{{\varvec{R}}}$**)** shown in Table S17 were calculated according to the equation reported by Janek et al.[62]. According to the results, after 10 µg mL^−1^ fsDNA immobilization, it was determined that ${{\varvec{\theta}}}_{{\varvec{I}}{\varvec{S}}}^{{\varvec{R}}}$ value was obtained as 0.313 on the surface of 2B/PPy/CG-SA cryogel/EDC-NHS electrodes. More repeatable results were obtained after EDC-NHS activation. Also, after fsDNA immobilization, it was obtained stable electrode surface.

### Analytical performance

#### Linear range and the limit of detection

The developed 2B/PPy/CG-SA cryogel electrodes were applied for the detection of fsDNA at concentrations ranging from 2.5 to 20 µg mL^−1^. The change observed in the average anodic current (∆Ia) after immobilization was observed to decrease linearly between 2.5 and 20 µg mL^−1^ when plotting against the concentration of fsDNA (Fig. S10). The values of ∆Ia are explained according to Eq. 3:

3 $$\Delta \text{Ia}={Ia}_{\text{fsDNA}}-{Ia}_{\text{EDC}/\text{NHS}}$$

${Ia}_{\text{fsDNA}}$ is the anodic peak current after fsDNA immobilization, and ${Ia}_{\text{EDC}/\text{NHS}}$ is the anodic peak current after EDC-NHS activation.

The highest value was recorded as ∆Ia 11.65 ± 1.07 µA (RSD%, 9.23%, *n* = 4) at 2.5 µg mL^−1^ concentration, while 3.73 ± 0.70 µA (RSD%, 18.91%, *n* = 4) at 20 µg mL^−1^ concentration was recorded as the lowest ∆Ia value. All results are presented in Table S18.

CVs (Fig. S9) confirmed that the values of the anodic peak current signal decreased as the fsDNA concentration increased. This was interpreted as immobilized fsDNA inhibited electron transfer at the electrode surface.

**Fig. 8:**
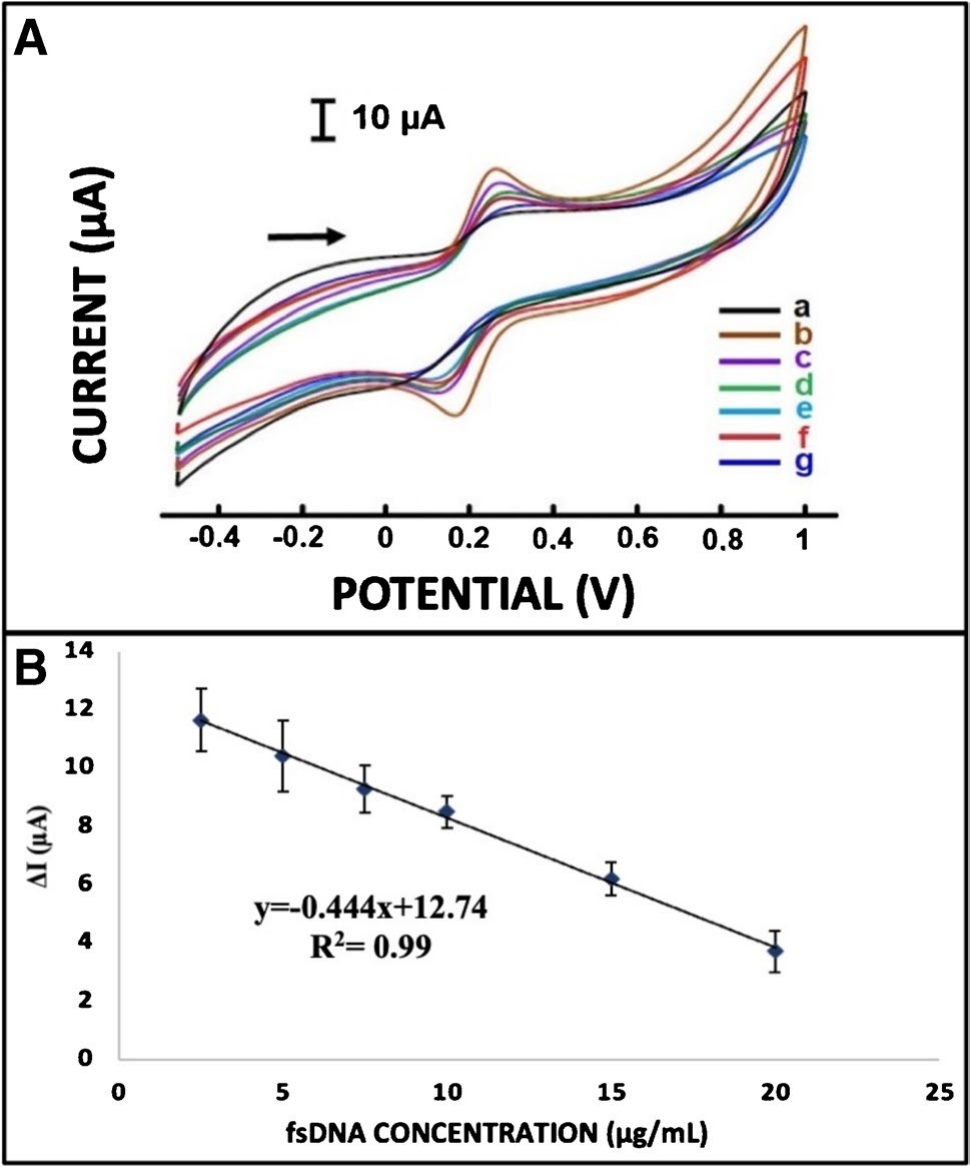
**A** Cyclic voltammograms of 2.5 mM redox probe solution at fsDNA immobilized modified electrodes (a) 2B/PPy/CG-SA cryogel/EDC-NHS modified electrode, (b) 2.5 µg mL^−1^ fsDNA, (c) 5 µg mL^−1^ fsDNA, (d) 7.5 µg mL^−1^ fsDNA, (e) 10 µg mL^−1^ fsDNA, (f) 15 µg mL^−1^ fsDNA, and (g) 20 µg mL^−1^ fsDNA. **B** Calibration graph obtained after immobilization of fsDNA at different concentrations on 2B/PPy/CG-SA cryogel/EDC-NHS electrodes in 2.5 mM redox probe solution

The electrochemical response, depending on the relationship between the DNA concentration and the change in oxidation currents, is explained by the calibration graph in Fig. 8. According to the Miller and Miller method [63], limit of detection (LOD) was calculated and found to be 0.98 µg mL^−1^ ((LOD = 3.3 × (standard deviation/the slope of the calibration curve)). The sensitivity of the developed DNA biosensor was calculated as 14.8 µA mM^−1^ cm^−2^.

The reproducibility of the cryogel polysaccharide-based 2B electrodes used was shown by repeated measurements on three different days after immobilization with 2.5 µg mL^−1^ fsDNA, and the % RSD value was calculated as 11.47 ± 0.89 µA (RSD % = 7.74%, *n* = 6).

#### Stability of the biosensor

Storage stability of 2B/PPy/CG-SA cryogel electrode tested for 45 days using CV technique. The modified electrodes were stored at + 4 °C for 1, 5, 14, 21, and 45 days, and electrochemical measurements were performed. On day 5, a decrease of 18.13% was observed in the peak current measured with electrodes compared to day 1. However, there was only 33.05% loss in response on the 45th day, in contrast to the response on the first day (Fig. S11). Under the better storage conditions (e.g., storage in blisters, protection from darkness and moisture, preservation), the stability of the 2B/PPy/CG-SA cryogel electrode could be better; however, it still provides easy to use application with cost-effectiveness for users.

## Conclusion

The polysaccharide-cryogel/polypyrrole-based electrode was introduced for the first time herein, and then, the voltammetric determination of DNA by PPy-cryogel–modified electrodes was demonstrated in this present study as the proof of concept.

The advantages of these electrodes are biocompatible and easily accessible since the polysaccharides used in cryogel synthesis are natural, and the modified electrodes developed are cost-effective since low-cost electrodes are used since the surface of 2B electrodes is modified. In comparison to existing studies in the literature, 2B/PPy/CG-SA cryogel electrodes offer advantages over other studies by enabling electrochemical DNA determination with short DNA immobilization time, low detection limit, short measurement time, and affordable cost [8, 10, 24, 38–40, 64–68] and also summarized in Table S19. Unlike the study of Liu et al. [38], conductive carrageenan and carrageenan-sodium alginate cryogel–modified electrodes were prepared in the present work for the first time in the literature and the prepared electrode surfaces were activated with EDC-NHS. Salahuddin et al. [39] reported a chronocoulometric determination of miRNA-9–2 using a κ-carrageenan hydrogel-coated mesoporous gold (Au) electrode after performing “magnetic ısolation and purification” process to obtain the sample of miRNA-9–2. In contrast to work of Salahuddin et al. [39] based on κ-carrageenan hydrogel modified gold electrode, 2B/PPy/CG-SA cryogel electrode was developed as disposable in our present work. Thus, the possible implementation of 2B/PPy/CG-SA cryogel electrode was presented herein for the development of single-use biosensor devices. In contrast to Congur et al. [40], the fsDNA immobilization time was completed only in 30 min, and accordingly, the determination of fsDNA by CV was performed in a shorter time with a lower detection limit (0.98 µg mL^−1^).

In contrast to the study reported by Esmaeili et al. [24], EDC-NHS was also used in this instance to activate the surface of cryogel-modified electrodes containing carrageenan and sodium alginate. As a result, the surface became more stable for DNA immobilization at the next step via covalent bonding of the -COOH groups of sodium alginate polysaccharide on the EDC-NHS activated electrode surface and the amine (-NH_2_) groups on the guanine base present in fsDNA [35, 43–46]. Moreover, electrochemical detection of DNA was performed 35 min after DNA immobilization using EDC-NHS activated surface of PPy/CG-SA cryogel–modified electrode.

In earlier studies in literature [54–58], the determination of different analytes such as sialic acid, prostate-specific antigen, glucose, uric acid, and carcinoembryonic antigen with cryogel-modified electrodes was investigated by using numerous working electrodes, such as glassy carbon electrodes, gold electrodes, and screen printed electrodes, which were more costly and time-consuming to prepare in contrast to the 2B graphite electrode used in our study.

Moreover, the cryogel support material developed in our study can be modified and new functional groups can be added to its structure. The structure can be designed analyte-specific for the analysis of different materials. Less material (monomer/polymer) is used in cryopolymerization. It is also possible to provide point of care conversion of this type of electrodes. Initially, the cryogel synthesis time of 48 h seemed to be a problem. This was overcome in our study by simultaneously modifying at least 60 electrodes prior to immobilization with fsDNA. fsDNA immobilization onto the surface of PPy-cryogel-modified electrodes is completed in less than 50 min. The storage stability of these electrodes was investigated, and it retained 66. 95% of its response up to 45th day.

The limit of detection (LOD) in the linear concentration range of 2.5–20 μg mL^−1^ on the surface of the developed modified electrodes was calculated as 0.98 μg mL^−1^ with 99% reliability. The sensitivity of DNA biosensor was found to be 14.8 µA mM^−1^ cm^−2^. The conductive polysaccharide-based cryogel-modified electrode has several advantages, such as easy preparation, cost-effectiveness, high biomolecule immobilization capacity, and conductive properties that enable sensitive electrochemical analysis of biomolecules.

The electrochemical detection of DNA by the conductive PPy-cryogel-modified electrodes was demonstrated in the present study as proof of concept. In our targetting studies, the PPy-cryogel-modified electrodes can be furtherly applied for the detection of DNA interaction with anticancer agent, or any of specific analytes (protein, toxin, etc.) after the selectivity of biosensor was tested in various sample matrices (urine, serum, saliva, etc.).

### Supplementary Information

Below is the link to the electronic supplementary material.

Supplementary file1 (DOCX 5.88 MB)

## Data Availability

Not applicable.

## Abbreviations

*CG*: Carrageenan
*SA*: Sodium alginate
*PPy*: Polypyrrole
*TGA*: Thermogravimetric analysis
*FTIR*: Fourier-transform infrared spectroscopy
*SEM*: Scanning electron microscopes
*EDX*: Energy-dispersive X-ray spectroscopy
*CV*: Cyclic voltammetry
*EIS*: Electrochemical impedance spectroscopy
*DNA*: Deoxyribonucleic acid
*fsDNA*: Fish sperm DNA
*GDE*: Glycerol diglycidyl ether
*EDC*: 1-Ethyl-3-(3-dimethylaminopropyl carboimide) hydrochloride
*NHS*: N-hydroxysuccinimide
*DW*: Double-distilled water
*SPE*: Screen-printed electrode
