# Supplementary material for: Synthesis and characterization of polysaccharide-cryogel and its application to the electrochemical detection of DNA
Source: Mikrochim Acta. 2024 Aug 1;191(8):499. doi: 10.1007/s00604-024-06550-7 (PMC11294392; doi:10.1007/s00604-024-06550-7)
Supplement: Supplementary file 1 — Supplementary file1 (DOCX 5.88 MB) [file 604_2024_6550_MOESM1_ESM.docx]

**Supplementary Materials**

**Synthesis and characterization of polysaccharide-cryogel and its application to the electrochemical detection of DNA**

**Nilay Tunca^1,2,3[0000-0003-4314-6096]^, Meltem Maral^2^**^[^**^0000-0002-0306-2181]^,** **Esma Yildiz^2[0000-0002-8094-8277]^,**

**Sultan Butun Sengel^3,**[0000-0001-7036-2224]^ and Arzum Erdem^2,*[0000-0002-4375-8386]^**

^1^The Institute of Natural and Applied Sciences, Biomedical Technologies Department, Ege University, Bornova, 35100 Izmir, Turkey

^2^Analytical Chemistry Department, Faculty of Pharmacy, Ege University, Bornova, 35100 Izmir, Turkey

^3^Faculty of Engineering and Architecture, Department of Biomedical Engineering, Eskisehir Osmangazi University, 26480 Eskisehir, Turkey

*** Corresponding author :** A. Erdem, [arzum.erdem@ege.edu.tr](mailto:arzum.erdem@ege.edu.tr)

**Co-corresponding author: S. Butun Sengel, [sultanbutun.sengel@ogu.edu.tr](mailto:sultanbutun.sengel@ogu.edu.tr)

Other author email:

Nilay Tunca: nilay_tnc@hotmail.com

Meltem Maral: meltemmaral58@gmail.com

Esma Yıldız: [esmayildiz94@gmail.com](mailto:esmayildiz94@gmail.com)

***To summarize the study in an outline;***

1) In order to observe the structures of the cryogels to be used for electrode modification, synthesis and characterization of CG cryogel, CG-SA cryogel, PPy/CG cryogel and PPy/CG-SA cryogel were carried out outside the electrode surface.

2) Based on the optimum conditions determined at the external cryogel synthesis stage; the optimum electrode composition was determined.

3) For optimum electrode composition conditions; the volume of cryogel solution dripped on the electrode surface, pyrrole monomer concentration, pyrrole loading time and cryogel solution concentration ratios were changed and analyzed by electrochemical method.

4) On the electrode surface prepared under the optimum conditions (electrode components: volume of cryogel solution dripped on the surface: 3 μL; pyrrole monomer concentration: 0.5-M; pyrrole loading time: 30 min; cryogel solution concentration: 1% (w/v)) EDC-NHS activation was performed.

5) The behavior of 2B/PPy/CG cryogel and 2B/PPy/CG-SA cryogel modified electrodes after EDC-NHS activation was compared by CV technique.

6) According to CV measurement results, it was determined that the surface of 2B/PPy/CG-SA cryogel electrodes became more stable after activation of EDC-NHS in the presence of sodium alginate polysaccharide.

7) The effect of EDC-NHS activation time on 2B/PPy/CG-SA cryogel modified electrodes was investigated for 15 and 30 minutes.

8) Before fsDNA immobilization; 5 μL volume of EDC-NHS solution was dropped on the surface of 2B/PPy/CG-SA cryogel electrodes and kept for activation for 15 minutes.

9) After 15 minutes of EDC-NHS activation, 5 μL volumes of fsDNA solutions prepared at different concentrations for immobilization of fsDNA were dripped onto electrode surfaces and kept for 30 minutes for immobilization.

10) CV and EIS measurements were performed with the electrodes after the immobilization period was completed.

#### *Optimization of the cryogel composition*

#### Different sets of experiments were created to determine the synthesis parameters of cryogels synthesized on the electrode surface. Cryogels with different structures were produced by varying the solution concentration, mixing ratio, crosslinker ratio and cryogelation time. The results of the change in solution concentration are presented in Table S1, the results of the cryogels prepared at different mixing ratios for the CG-SA solution are presented in Table S2, the results of the cryogels synthesized with different cross-linker ratios are presented in Table S3 and the results of the cryogels synthesized at different cryogelation times are presented in Table S4.

**Table S1.** Properties of CG cryogels synthesized at different concentrations.

| CG solution concentration (mg mL^-1^) | Crosslinker ratio (%) | Cryogelation time (h) | Property of cryogel |
| --- | --- | --- | --- |
| 5.00 | 20 | 48 | Porous, spongy but not-elastic |
| 7.5 | 20 | 48 | Porous, spongy but not-elastic |
| 10.0 | 20 | 48 | Porous, spongy and keeps its shape |

**Table S2.** Properties of CG cryogels synthesized at different crosslinker ratios.

| CG solution concentration (mg mL^-1^) | Crosslinker ratio (%) | Cryogelation time (h) | Property of cryogel |
| --- | --- | --- | --- |
| 10.0 | 10 | 48 | Not porous and spongy |
| 10.0 | 20 | 48 | Porous and spongy |
| 10.0 | 40 | 48 | Porous, spongy but brittle |

**Table S3.** Properties of CG-SA cryogels prepared in different ratios of CG-SA solution mix.

| CG:SA solution mix ratio (v:v) | CG solution concentration (mg mL^-1^) | Crosslinker ratio (%) | Cryogelation time (h) | Property of cryogel |
| --- | --- | --- | --- | --- |
| 1:1 | 10.0 | 20 | 48 | Porous, spongy and keeps its shape |
| 2:1 | 10.0 | 20 | 48 | Porous, Brittle |
| 3:1 | 10.0 | 20 | 48 | Porous, Brittle |

**Table S4.** Properties of CG-SA cryogels produced at different cryogelation times.

| CG:SA solution mix ratio (v:v) | CG solution concentration (mg mL^-1^) | Crosslinker ratio (%) | Cryogelation time (h) | Property of cryogel |
| --- | --- | --- | --- | --- |
| 1:1 | 10.0 | 20 | 24 | Does not keep its shape |
| 1:1 | 10.0 | 20 | 48 | Porous and spongy structure |

**Table S5.** Optimum conditions determined for electrode composition

| **Variable** | **Optimum condition** |
| --- | --- |
| CG solution concentration | **10.0 mg mL^-1^** |
| CG:SA solution mix ratio | **1:1 (v:v)** |
| Crosslinker ratio | **20%** |
| Cryogelation time | **48 h** |


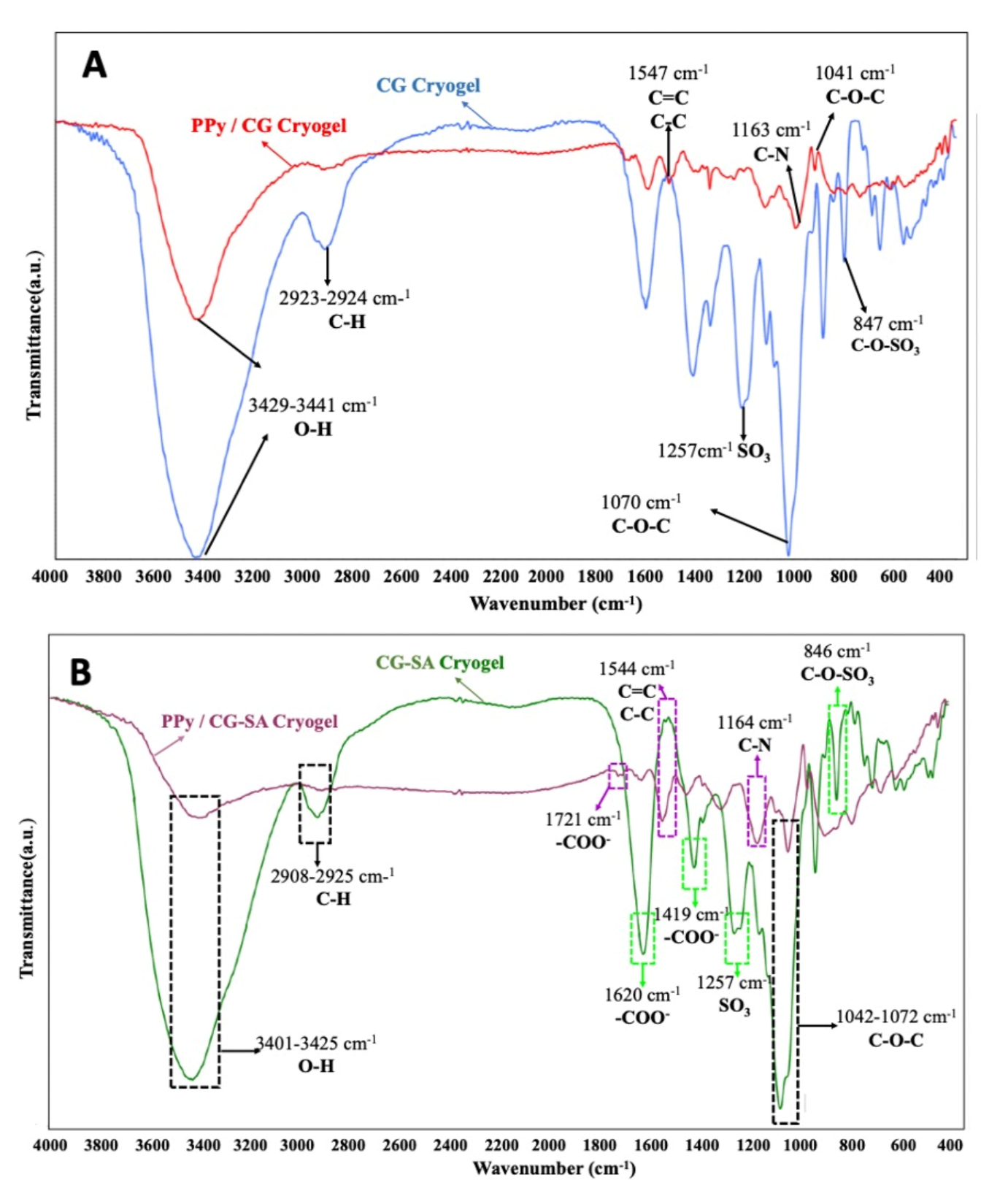


**Fig. S1.** FTIR spectra of the **(A)** CG cryogel, PPy / CG cryogel and **(B)** CG-SA cryogel, PPy / CG-SA cryogel.

**
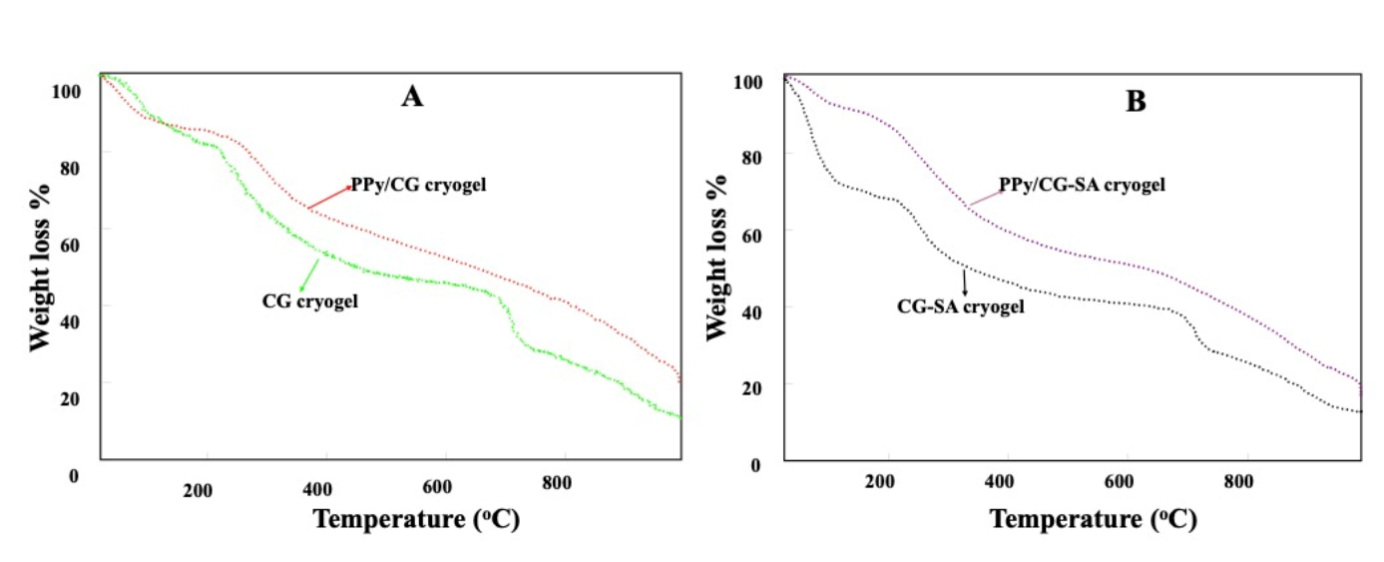
**

**Fig. S2.** Thermal gravimetric analysis (TGA) of **(A)** CG cryogel and PPy/CG cryogel, **(B)** CG-SA cryogel and PPy/CG- SA cryogel.

According to the TGA results, for the CG cryogel, a 25% weight loss was observed between 253-476 °C and a 21% weight loss between 476-760 °C. The residual mass at 900 °C was determined as 18% for CG cryogel. In the PPy / CG cryogel, a weight loss of 23% was recorded at 257-470 °C and a weight loss of 16% at 470-763 °C. When the temperature increased to 900 °C, the residual mass was determined as 32% for the PPy/CG cryogel. The thermal stability of carrageenan cryogels was concluded to increase after polypyrrole synthesis.

#### When the temperature-dependent weight change of CG-SA cryogel was examined, a weight loss of 19% was observed between the temperatures of 249-484 °C, and a mass loss of 15% between the temperatures of 484-762 °C. When the temperature increased to 800 °C, 25% of its mass remained intact. When the PPy / CG-SA TGA analyzes were evaluated, a 20% mass loss was observed between 252-465 °C and 15% between 465-760 °C temperatures. When the temperature was 800 °C, 38% of its mass remained intact.

*The electrochemical optimization of volume of solution dripped onto the electrode surface*

The effect of the volume of CG cryogel synthesized on the electrode surface on the electrode response was investigated. The cryogel was synthesized by dropping 1 μL, 2 μL, 3 μL and 4 μL volumes of cryogel solution at a concentration of 10.0 mg mL^-1^ onto the electrode surface, and then pyrrole was loaded into CG cryogels in 0.5-M, 0.5-mL aqueous pyrrole solution for 30 minutes. Then, CV measurements of the modified electrodes were performed. The results obtained are shown in Fig. S3. Average anodic peak current (Ia) and cathodic peak current (Ic), anodic relative charge (Qa) and cathodic relative charge (Qc), and calculated electroactive surface area (A) data were obtained for electrodes with different volumes of cryogel solution on their surfaces as shown in Table S6. The average values of Ia increased for all modified electrodes (Table S7).

**Table S6.** The average anodic peak current (Ia) cathodic peak current (Ic) anodic relative charge (Qa) cathodic relative charge (Qc) and the calculated electroactive surface area values (A) of 2B/PPy/CG cryogel modified electrodes prepared at 1 μL, 2 μL, 3 μL and 4 μL volumes (n=2).

| **Electrodes** | **Ia (μA),**  **RSD%** | **Ic (μA),**  **RSD%** | **Qa (mC), RSD%** | **Qc (mC),**  **RSD%** | **A (cm^2^)** |
| --- | --- | --- | --- | --- | --- |
| **2B** | 5.42 ± 0.84,  15.59% | 4.65 ± 0.68,  14.75% | 0.19 ± 0.01,  3.82% | 0.33 ± 0.02,  6.53% | 0.016 |
| **1 μL 2B/PPy/CG cryogel** | 11.75 ± 8.41,  71.60% | 16.46 ± 3.29,  19.94% | 1.48 ± 0.84,  56.38% | 2.01 ± 1.06,  52.69% | 0.035 |
| **2 μL 2B/PPy/CG cryogel** | 15.50 ± 0.81,  5.22% | 15.85 ± 2.92,  18.23% | 2.91 ± 0.94,  32.30% | 3.51 ± 1.29,  36.76% | 0.046 |
| **3 μL 2B/PPy/CG cryogel** | 15.05 ± 0.50,  3.30% | 15.63 ± 1.63,  10.48% | 3.72 ± 2.37,  63.76% | 4.83 ± 3.60,  74.55% | 0.045 |
| **4 μL 2B/PPy/CG cryogel** | 11.46 ± 5.19,  45.22% | 13.35 ± 3.25,  24.37% | 2.86 ± 1.52,  53.30% | 4.44 ± 3.25,  73.23% | 0.034 |

**Table S7.** The change of the average Ia value of the electrodes prepared at different solution volume according to the 2B electrode (n=2).

| **Volume of solution** | **Increase of Ia (%)** |
| --- | --- |
| 1 μL | 53.9 |
| 2 μL | 65.0 |
| 3 μL | 64.0 |
| 4 μL | 52.7 |

The highest increase was observed at electrodes with cryogel volumes of 2 μL (65.0%) and 3 μL (64.0%). It was observed that the increase in the anodic peak current values of the 2B/PPy/CG cryogel modified electrodes with polymeric film volumes of 2 μL and 3 μL were close to each other. Since the signals measured at 2B/PPy/CG cryogel electrodes with cryogel solution volume of 3 μL were more reproducible (RSD %, 3.30%, n=2), the optimum volume of cryogel solution dripped onto the surface was determined as 3 μL.


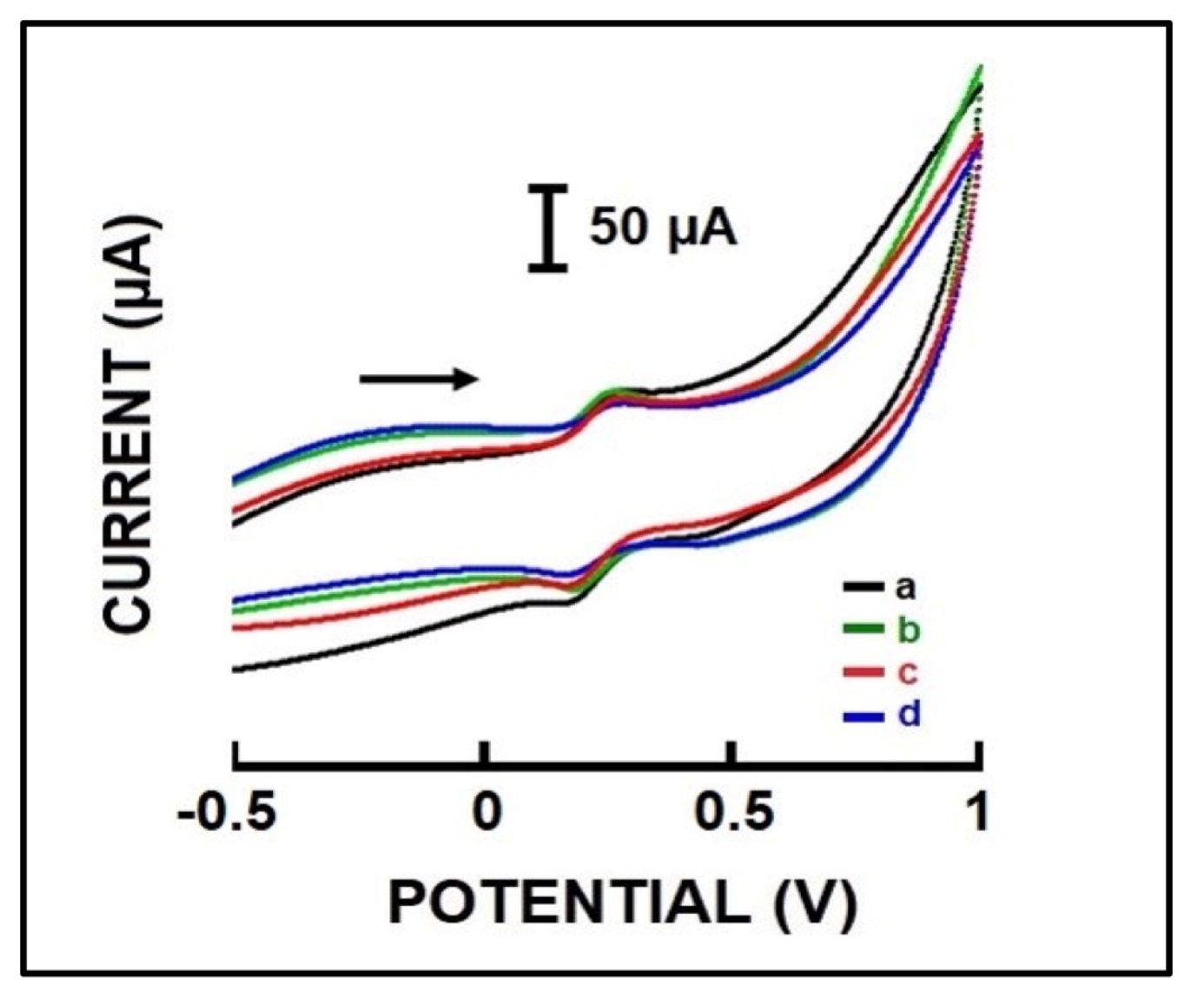


**Fig. S3.** Cyclic voltammograms of 2.5 mM redox probe solution at 2B/PPy/CG cryogel modified electrodes prepared by dropping different volumes of cryogel solution (a) 1 μL (b) 2 μL (c) 3 μL (d) 4 μL.

#### *The electrochemical optimization of PPy monomer concentration*

For the pyrrole concentration optimization study, the concentration of the CG cryogel solution on the 2B electrode surfaces was kept constant at 10.0 mg mL^-1^, the pyrrole loading time was 30 minutes, and the solution volume was 3 μL. Polypyrrole conductive polymer was synthesized on the 2B/CG cryogel electrode with pyrrole solutions prepared as 0.25 M, 0.5 M and 0.75 M. CV measurements were performed with electrodes prepared at different concentrations of pyrrole monomer (Fig. S4). For electrodes with different concentrations of pyrrole, the average anodic peak current (Ia) and cathodic peak current (Ic), the relative anodic charge (Qa), the relative cathodic charge (Qc) and the electroactive surface area (A) data are presented in Table S8.

The increase in anodic peak current values at modified electrodes prepared with 0.25, 0.5 and 0.75 M pyrrole solution was calculated as 53.9%, 65.4% and 62.1%, respectively. The highest increase was seen in the electrodes with a concentration of 0.5 M pyrrole solution. Therefore, the optimum concentration of pyrrole solution was determined as 0.5 M.

**Table S8.** The average anodic peak current (Ia) cathodic peak current (Ic) anodic relative charge Qa cathodic relative charge Qc and the calculated electroactive surface area values (A) of 2B/PPy/CG cryogel modified electrodes prepared with 0.25 M, 0.5 M and 0.75 M pyrrole solutions (n=2).

| **Electrodes** | **Ia (μA),**  **RSD%** | **Ic (μA),**  **RSD%** | **Qa (mC),**  **RSD%** | **Qc (mC),**  **RSD%** | **A (cm^2^)** |
| --- | --- | --- | --- | --- | --- |
| **2B** | 5.42 ± 0.84,  15.59% | 4.65 ± 0.68,  14.75% | 0.19 ± 0.01,  3.82% | 0.33 ± 0.02,  6.53% | 0.016 |
| **0.25 M 2B/PPy/CG cryogel** | 11.77 ± 5.59,  47.55% | 16.88 ± 3.9,  23.11% | 1.29 ± 0.27,  20.83% | 1.37 ± 0.05,  3.63% | 0.035 |
| **0.5 M 2B/PPy/CG cryogel** | 15.65 ± 0.03,  0.19% | 16.58 ± 0.49,  2.92% | 2.43 ± 0.62,  25.37% | 2.80 ± 0.11,  4.04% | 0.047 |
| **0.75 M 2B/PPy/CG cryogel** | 14.29 ± 1.66,  11.66% | 15.81 ± 3.71,  23.65% | 2.83 ± 1.59,  56.32% | 4.25 ± 2.44,  57.47% | 0.043 |


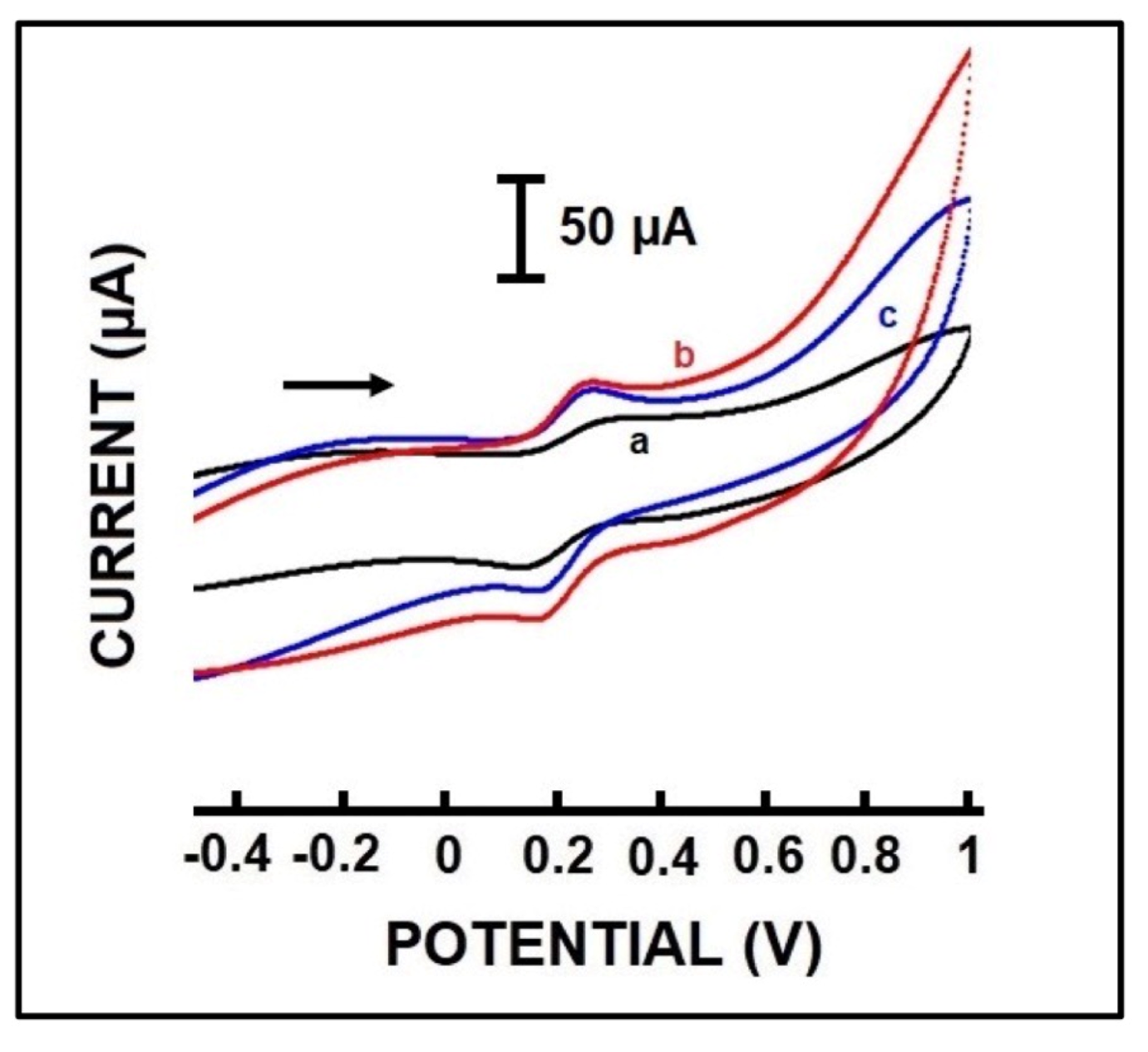


**Fig. S4.** Cyclic voltammograms of 2.5 mM redox probe solution at 2B/PPy/CG cryogel modified electrodes prepared with different pyrrole solution concentration (a) 0.25 M (b) 0.5 M (c) 0.75 M.

#### *The electrochemical optimization of PPy synthesis reaction time*

10.0 mg mL^-1^ cryogel solution concentration, 3 μL cryogel solution volume and 0.5 M pyrrole monomer concentration remained constant and the effect of PPy synthesis time was investigated. Polypyrrole synthesis was carried out for 15, 30, 45 and 60 min. on the electrodes whose cryogelation process was completed. The behavior of the modified electrodes prepared at different synthesis times was investigated by the cyclic voltammetry technique (Fig. S5). Electrodes prepared at different PPy synthesis times, average anodic peak current (Ia) and cathodic peak current (Ic), anodic relative charge (Qa), cathodic relative charge (Qc) and electroactive surface area (A) data are given in Table S9.

When the CV measurement results of the modified electrodes were compared with the 2B electrode, an increase in the average Ia values of all was observed (Table S10).

**Table S9.** The average values (n=2) of anodic (Ia) and cathodic (Ic) peak current and anodic relative charge (Qa) and cathodic relative charge (Qc) and electroactive electrode surface (A) measured with electrodes modified at different PPy synthesis times.

| **Electrodes** | **Ia (μA),**  **RSD %** | **Ic (μA),**  **RSD %** | **Qa (mC), RSD%** | **Qc (mC), RSD%** | **A (cm^2^)** |
| --- | --- | --- | --- | --- | --- |
| **2B** | 5.42 ± 0.84,  15.59% | 4.65 ± 0.68,  14.75% | 0.19 ± 0.01,  3.82% | 0.33 ± 0.02,  6.53% | 0.016 |
| **15 minutes**  **2B/PPy/CG cryogel** | 9.62 ± 4.98,  51.73% | 15.64 ± 3.89,  24.89% | 0.86 ± 0.25,  29.60% | 1.29 ± 0.15,  11.56% | 0.029 |
| **30 minutes**  **2B/PPy/CG cryogel** | 15.65 ± 0.03,  0.19% | 16.58 ± 0.49,  2.92% | 2.43 ± 0.62  25.37% | 2.80 ± 0.11,  4.04% | 0.047 |
| **45 minutes**  **2B/PPy/CG cryogel** | 16.33 ± 2.12,  13.02% | 18.02 ± 0.70,  3.90% | 2.45 ± 0.67,  27.47% | 2.80 ±1.29,  46.30% | 0.049 |
| **60 minutes**  **2B/PPy/CG cryogel** | 15.67 ± 0.08,  0.55% | 18.30 ± 2.67,  14.63% | 1.56 ± 0.57,  36.26% | 1.36 ± 0.47,  34.96% | 0.047 |

**Table S10.** The change of the average Ia value of the electrodes prepared at different PPy synthesis times compared to the 2B electrode and the RSD % values (n=2).

| **PPy synthesis reaction time** | **Increase of Ia (fold) and RSD %** |
| --- | --- |
| 15 minutes | 1.77, 51.73% |
| 30 minutes | 2.88, 0.19% |
| 45 minutes | 3.01, 13.2% |
| 60 minutes | 2.89, 0.55% |

When the increase values in the signals were examined, it was seen that the values in the synthesis times of 30, 45 and 60 minutes were close to each other. When the results were evaluated in terms of reproducibility, it was determined that the best relative standard deviation value belonged to the electrodes with a synthesis time of 30 minutes (RSD%, 0.19%, n=2). The optimum PPy synthesis time was determined as 30 minutes, taking into account the cryogel solution volume and pyrrole monomer concentration optimization studies.


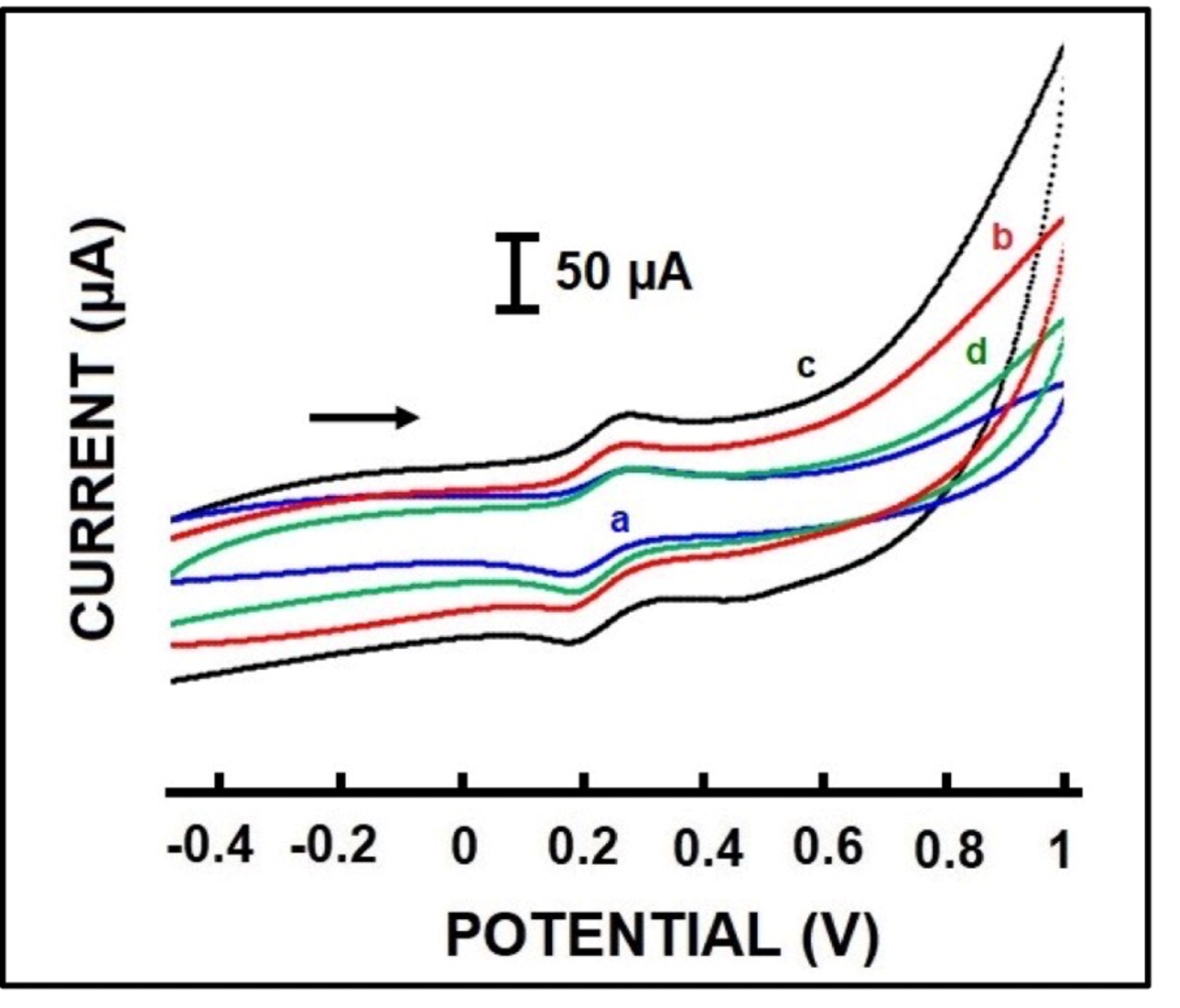


**Fig. S5.** Cyclic voltammograms of 2.5 mM redox probe solution at 2B/PPy/CG cryogel modified electrodes prepared at different PPy synthesis times (a) 15 minutes (b) 30 minutes (c) 45 minutes (d) 60 minutes.

#### *The electrochemical optimization of cryogel solution concentration*

3 µL of CG solutions prepared at concentrations of 15.0, 10.0, 5.0 mg mL^-1^ were dropped onto the surface of the 2B electrode. CV measurements of the modified electrodes, which were kept at −20°C for 48 h for cryogel synthesis, and then PPy synthesis was performed in 0.5 M 0.5 L aqueous pyrrole solution for 30 minutes. The results obtained are shown in Figure S6. Among electrodes prepared at different concentrations of CG solution, the average values of anodic peak current (Ia) and cathodic peak current (Ic), anodic relative charge (Qa), cathodic relative charge (Qc) and electroactive surface area (A) are shown in Table S11.

When the obtained data were evaluated, no peak current produced by the electrode was observed in the measurements performed inside the redox probe only in the electrodes modified with CG cryogel. This response was explained by the non-conductive nature of CG cryogels reducing electron transfer. Compared to the 2B electrode and the 2B/CG cryogel modified electrode, an increase in the anodic currents of the modified electrodes was observed after the 2B/PPy/CG cryogel modification (Table S12). This result showed that PPy incorporated into the electrodes after 2B/PPy/CG cryogel modification significantly improved the conductivity and the PPy conductive polymer was synthesized inside the CG cryogels.

**Table S11.** Average anodic peak current (Ia) cathodic peak current (Ic) anodic relative charge Qa cathodic relative charge Qc and calculated electroactive surface area values (A) of electrodes modified with 2B/PPy/CG cryogel prepared with cryogel solutions of 15.0, 10.0, 5.0 mg mL^-1^ (n = 2).

| **Electrodes** | **Ia (μA),**  **RSD%** | **Ic (μA),**  **RSD%** | **Qa (mC),**  **RSD%** | **Qc (mC), RSD%** | **A (cm^2^)** |
| --- | --- | --- | --- | --- | --- |
| **2B** | 5.42 ± 0.84,  15.59% | 4.65 ± 0.68,  14.75% | 0.19 ± 0.01,  3.82% | 0.33 ± 0.02,  6.53% | 0.016 |
| **15.0 mg mL^-1^**  **2B/PPy/CG cryogel** | 15.83 ±0.76,  4.77% | 18.90 ±0.40,  2.13% | 1.83 ±0.44,  23.96% | 1.71 ±0.11,  6.62% | 0.047 |
| **10.0 mg mL^-1^**  **2B/PPy/CG cryogel** | 20.27 ±6.29,  31.05% | 9.75 ±0.33,  3.37% | 3.87 ±0.82,  21.19% | 4.74 ±1.01,  21.36% | 0.061 |
| **5.0 mg mL^-1^**  **2B/PPy/CG cryogel** | 14.77 ±0.05,  0.34% | 15.32 ±2.19,  14.31% | 2.38 ± 0.63,  26.50% | 2.78 ± 0.80,  28.79% | 0.044 |

**Table S12.** The change of the average Ia value of the electrodes prepared at different cryogel solution concentration (mg mL^-1^) compared to the 2B electrode (n=2).

| **Cryogel solution concentration (mg mL^-1^)** | **Increase of Ia (%)** |
| --- | --- |
| 15.0 | 65.8 |
| 10.0 | 73.3 |
| 5.0 | 63.3 |

It was concluded that the highest increase in the signals was obtained with 10.0 mg mL^-1^ modified electrodes. Therefore, the optimum concentration was determined as 10.0 mg mL^-1^.


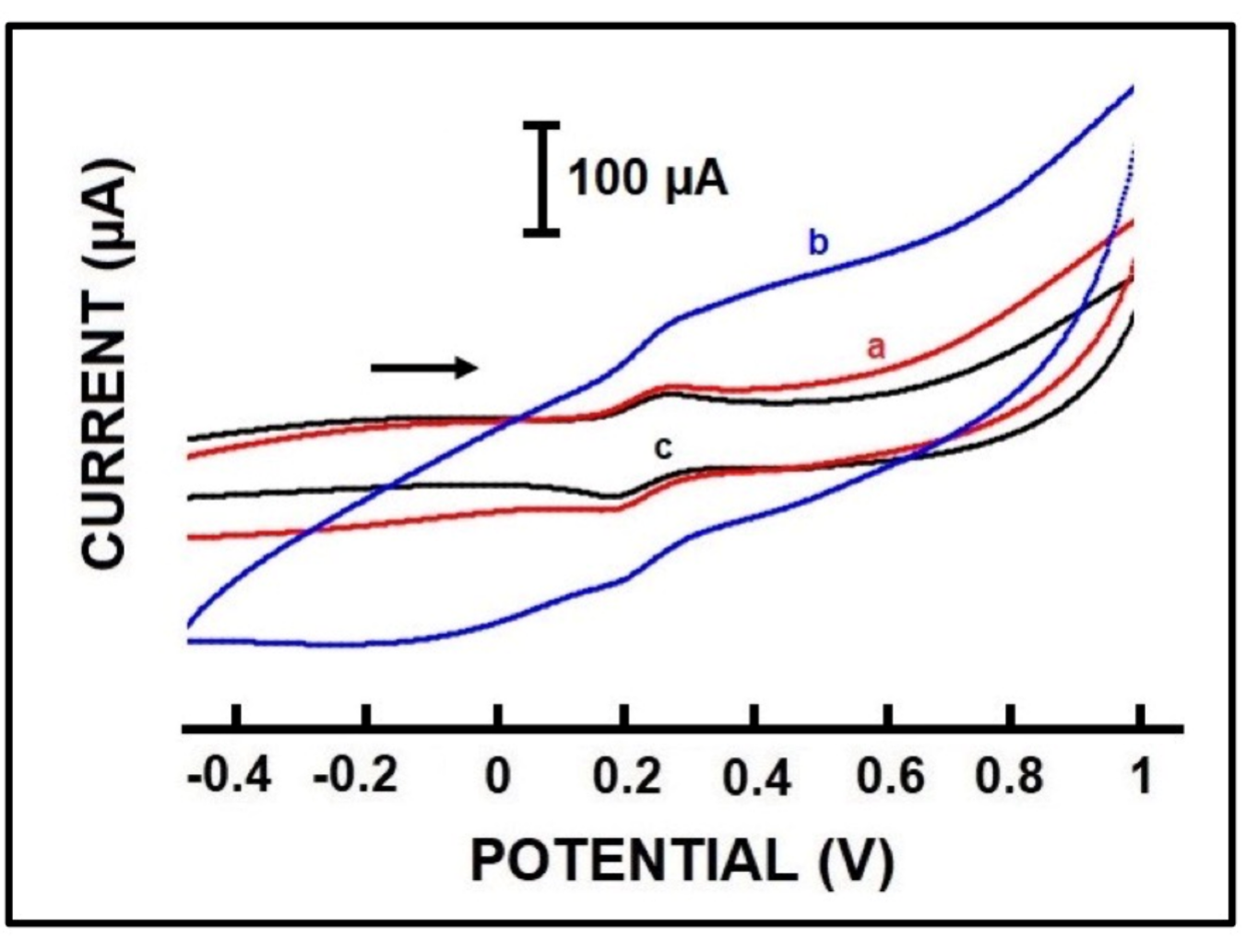


**Fig S6.** Cyclic voltammograms of 2.5 mM redox probe solution at 2B/PPy/CG cryogel modified electrodes prepared at different concentrations of CG (a) 5.0 mg mL^-1^ (b) 10.0 mg mL^-1^ (c) 15.0 mg mL^-1^.

The optimum conditions determined for the 2B/PPy/CG cryogel electrode composition as a result of all optimization studies are shown in Table S13.

**Table S13.** Optimum conditions determined for electrode composition.

| **Parameters** | **Tested Range** | **Selected Value** |
| --- | --- | --- |
| **CG-cryogel solution volume** | 1-2-3-4 μL | 3 μL |
| **Pyrrole monomer molarity** | 0.25-0.5-0.75 M | 0.5 M |
| **PPy synthesis time** | 15-30-45-60 min | 30 min |
| **CG solution concentration** | 5.0-10.0-15.0 mg mL^-1^ | 10.0 mg mL^-1^ |


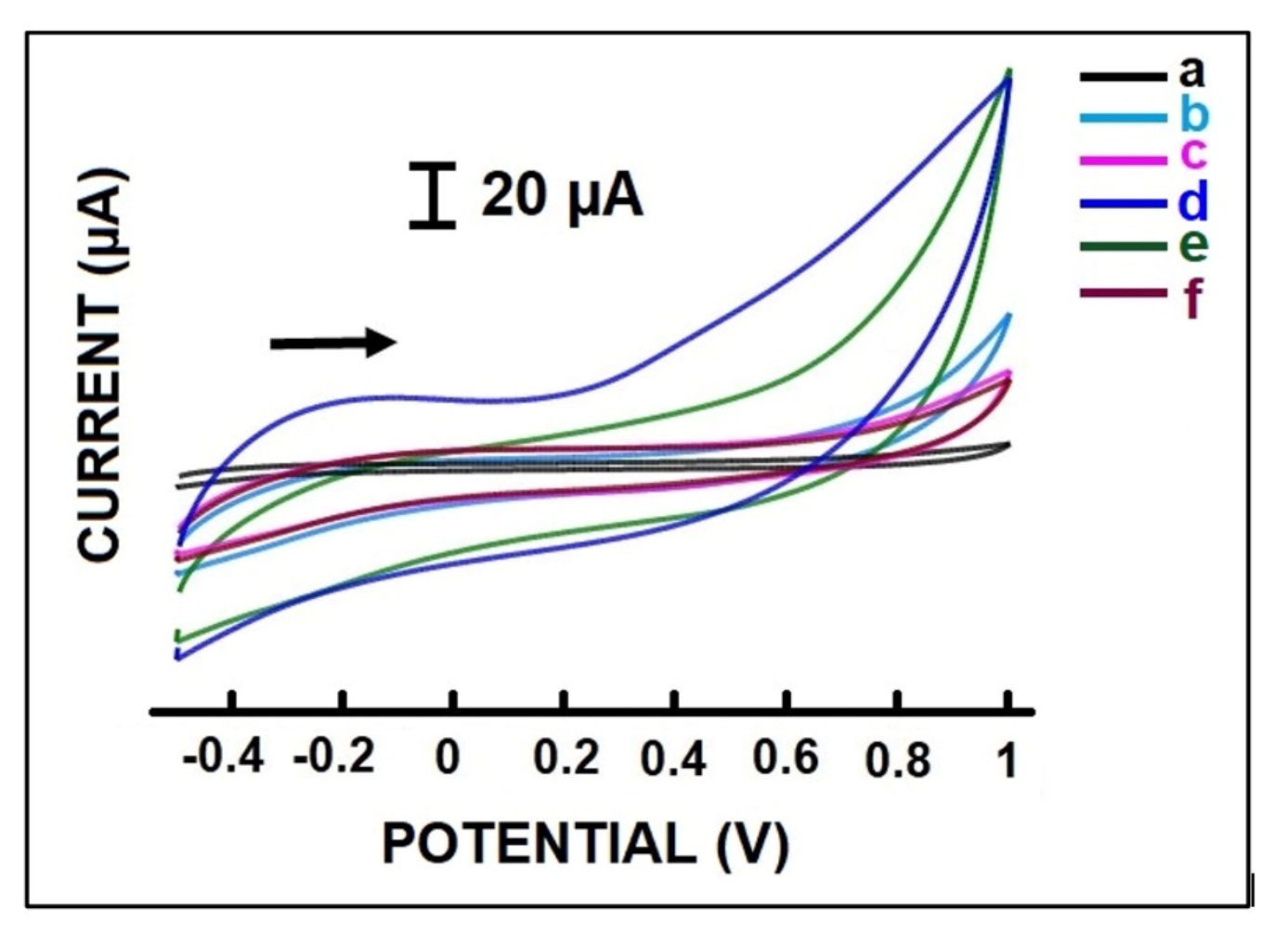


**Fig. S7.** Cyclic voltammograms of 0.1 M KCl solution at (a) 2B, (b) 2B/SA, (c) 2B/CG cryogel, (d) 2B/CG/SA cryogel, (e) 2B/PPy/CG cryogel, (f) 2B/PPy/CG-SA cryogel electrodes.

**Table S14.**  The average anodic peak current (Ia) cathodic peak current (Ic) anodic relative charge Qa, cathodic relative charge Qc, RSD % and surface area values (A) for 2B, 2B/SA, 2B/CG cryogel, 2B/CG/SA cryogel, 2B/PPy/CG cryogel, 2B/PPy/CG-SA cryogel electrodes (n=2).

| **Electrodes** | **Ia (µA),**  **RSD%** | **Ic (µA),**  **RSD%** | **Qa (mC),**  **RSD%** | **Qc (mC),**  **RSD%** | **A (cm^2^)** |
| --- | --- | --- | --- | --- | --- |
| **2B** | 3.28 ± 0.14,  4.39% | 4.10 ± 0.09,  2.38% | 0.26 ± 0.14,  54.39% | 0.19 ± 0.07,  37.22% | 0.009 |
| **2B/ SA** | No signal | No signal | 1.09 ± 0.04  3.89% | 0.52 ± 0.07  13.60% | - |
| **2B/CG cryogel** | No signal | No signal | 1.33 ± 0.45  34.03% | 0.64 ± 0.11  17.68% | - |
| **2B/CG/SA cryogel** | No signal | No signal | 1.40 ± 0.30  21.80% | 0.66 ± 0.25  37.78% | - |
| **2B/PPy/CG cryogel** | 12.64 ± 0.66  5.25% | 15.77 ± 0.47  3.00% | 0.69 ± 0.16  22.55% | 0.63 ± 0.10  15.71% | 0.038 |
| **2B/PPy/CG-SA cryogel** | 10.26 ± 1.27  12.43% | 11.90 ± 1.21  10.18% | 0.61 ± 0.01  1.17% | 0.69 ± 0.01  2.05% | 0.030 |

**Table S15.** Changes in Ia, RSD % and average Ia values for cryogel electrodes 2B/PPy/CG cryogel and 2B/PPy/CG-SA cryogel after activation of EDC-NHS and after immobilization of fsDNA (n = 2).

| **Electrodes** | **Ia (µA) with RSD%** | **% change in average current value** |
| --- | --- | --- |
| **2B/PPy/CG cryogel** | 12.64 ± 0.66, 5.25% |  |
| **2B/PPy/CG-SA cryogel** | 10.26 ± 1.27, 12.43% |  |
| **2B/PPy/CG-SA cryogel/fsDNA** | 9.47 ± 2.03, 21.48% | 7.70% decrease |
| **2B/PPy/CG cryogel-EDC-NHS** | 7.51 ± 5.66, 75.35% |  |
| **2B/PPy/CG-SA cryogel/EDC-NHS** | 10.14 ± 1.05, 10.38% |  |
| **2B/PPy/CG-SA cryogel/EDC-NHS/fsDNA** | 14.10 ± 0.89, 6.30% | 39.05% increase |

On the surface of the cryogel film containing sodium alginate polysaccharide, -COOH groups are present. When fsDNA is added to the EDC/NHS-activated surface, a covalent bond is formed between the amine group (-NH_2_) and the -COOH group on the guanine base of fsDNA.  In DNA immobilisation using reagents such as EDC/NHS, carboxyl groups on the surface are activated with the help of EDC/NHS reagents and then DNA molecules bind to these activated groups via amine groups. Thus, DNA is more stably bound to the electrode surface.


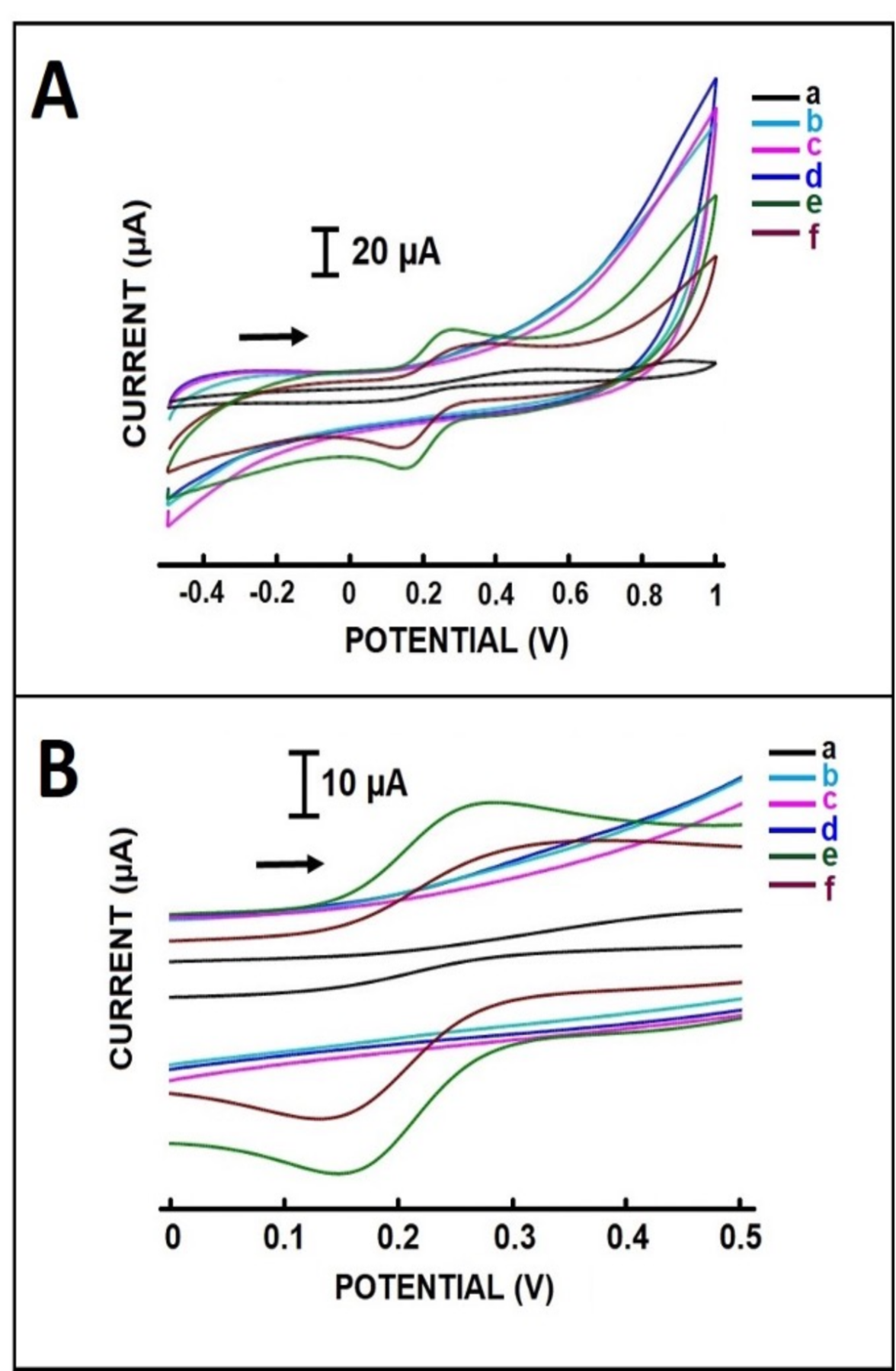


**Fig. S8.** Cyclic voltammograms of 2.5 mM redox probe solution at (a) 2B, (b) 2B/SA, (c) 2B/CG cryogel, (d) 2B/CG/SA cryogel, (e) 2B/PPy/CG cryogel, (f) 2B/PPy/CG-SA cryogel electrodes, Zoomed in view the anodic cathodic peak zone of the cyclic voltammograms of Fig 5.

**Table S16.**The avarege anodic peak current (Ia) cathodic peak current (Ic) anodic relative charge Qa cathodic relative charge Qc with RSD % values for 2B/PPy/CG-SA cryogel, 2B/PPy/CG/SA-cryogel/EDC-NHS and 2B/PPy/CG/SA-cryogel/EDC-NHS/fsDNA electrodes (n=3).

| **Electrodes** | **Ia (µA)**  **RSD%** | **Ic (µA)**  **RSD%** | **Qa (mC)**  **RSD%** | **Qc (mC)**  **RSD%** |
| --- | --- | --- | --- | --- |
| **2B/PPy/CG-SA cryogel** | 10.26 ± 1.27  12.43% | 11.90 ± 1.21  10.18% | 0.61 ± 0.01  1.17% | 0.69 ± 0.01  2.05% |
| **2B/PPy/CG-SA cryogel/EDC-NHS** | 5.89 ±0.60  10.15% | 7.96 ± 6.32  79.43% | 0.69 ± 0.35  50.71% | 0.60 ±0.21  35.13% |
| **10 µg/mL fsDNA immobilized**  **2B/PPy/CG-SA cryogel/EDC-NHS** | 14.13 ± 0.54  3.85% | 13.20 ± 4.08  30.90% | 0.64 ± 0.19  29.78% | 0.68 ±0.20  30.19% |
| **50 µg/mL fsDNA immobilized**  **2B/PPy/CG-SA cryogel/EDC-NHS** | 7.63 ± 1.77  23.13% | 6.02 ± 1.42  23.69% | 0.45 ± 0.12  26.68% | 0.49 ± 0.14  27.97% |


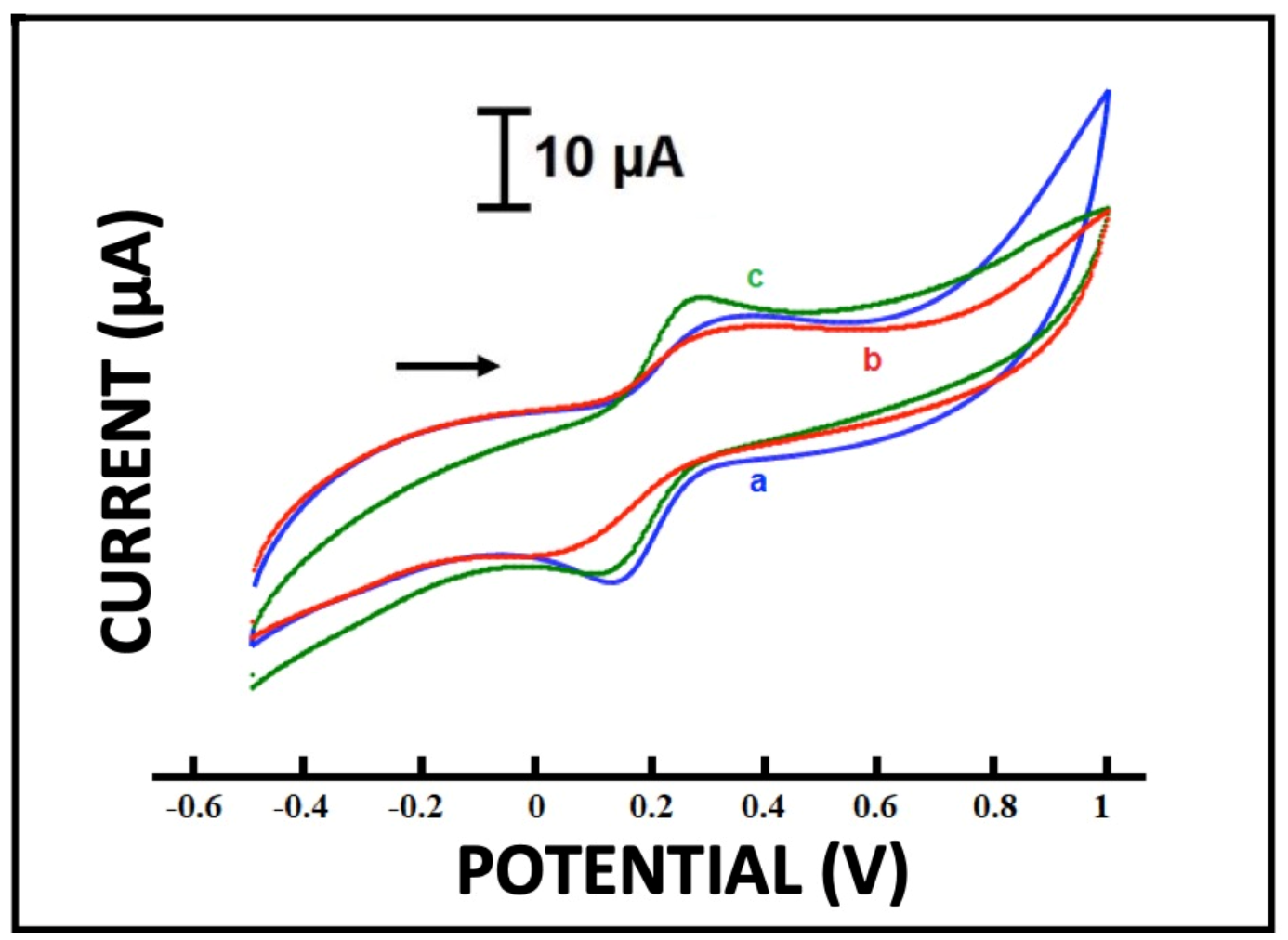


**Fig. S9**. Cyclic voltammograms of 2.5 mM redox probe solution at 2B/PPy/CG-SA cryogel modified electrodes activated with EDC-NHS a) EDC-NHS activation for 15 minutes b) EDC-NHS activation for 30 minutes.

**Table S17.** The average values of *Rct* presented with the % RSD values, the values of change in charge transfer resistance after activation of EDC-NHS and immobilization of fsDNA and the apparent fractional coverage value () after fsDNA immobilization (n=2).


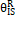

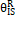


| ***Electrodes*** | ***The average values of R_ct_ (Ω) with RSD%*** | ***% increase in R_ct_*** | $\boldsymbol{\theta}_{\boldsymbol{IS}}^{\boldsymbol{R}}$ |
| --- | --- | --- | --- |
| ***2B/PPy/CG-SA cryogel*** | *201 ± 101,*  *50.43%* | *74.81%* | *0.428* |
| ***10 µg/mL fsDNA immobilized 2B/PPy/CG-SA cryogel*** | *351 ± 122,*  *34.90%* |  |  |
| ***2B/PPy/CG-SA cryogel/ EDC-NHS*** | *396 ± 110,*  *27.86%* | *45.58%* | *0.313* |
| ***10 µg/mL fsDNA immobilized 2B/PPy/CG-SA cryogel/EDC-NHS*** | *577 ± 66,*  *11.41%* |  |  |


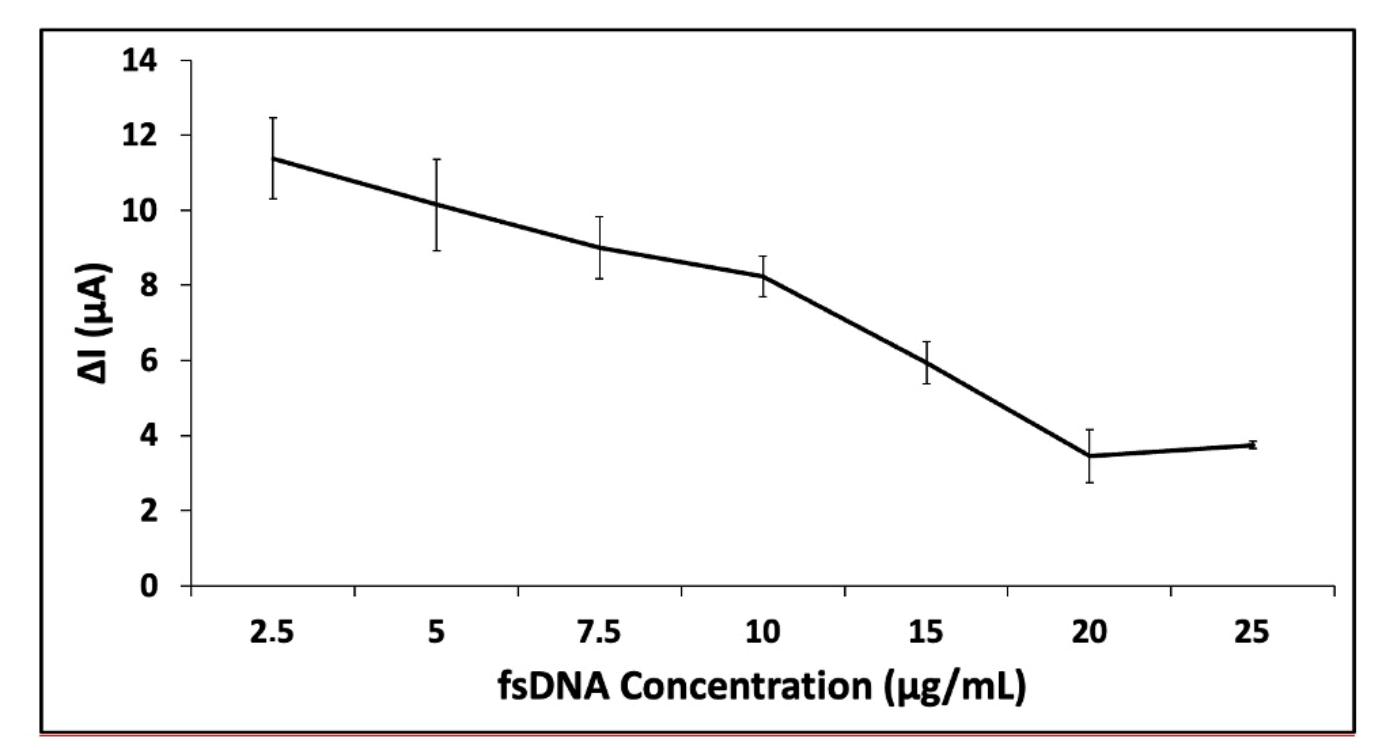


**Fig. S10.** Line graph presenting the average current values measured after immobilization of the fsDNA concentration range of 2.5-25 µg/mL using 2B/PPy/CG-SA cryogel/EDC-NHS electrodes (n=4).

The reproducibility of the developed DNA biosensor was analysed on three different days and the results are presented in Table S19 of the revised supp. Material. The relative standard deviation (RSD%) was calculated based on the results in different concentrations of fsDNA that were obtained in three different days. Accordingly, the RSD value was found to be ≤ 10% each fsDNA concentration. It can be concluded that PPy/CG cryogel modified electrode provides sensitive and responsive analysis for voltammetric detection of DNA.

***Table S19.*** *The average* **ΔIa values** *with RSD (%) (n=3) obtained in presence of fsDNA in different concentrations.*

| **fsDNA concentrations (µg mL^-1^)** | **Average ΔIa values (μA)** | **RSD (%)** |
| --- | --- | --- |
| 2.5 | 11.48 ± 0.33 | 2.88 |
| 5 | 10.93 ± 0.81 | 7.49 |
| 7.5 | 9.36 ± 0.97 | 10.44 |
| 10 | 8.85 ± 0.70 | 8.01 |


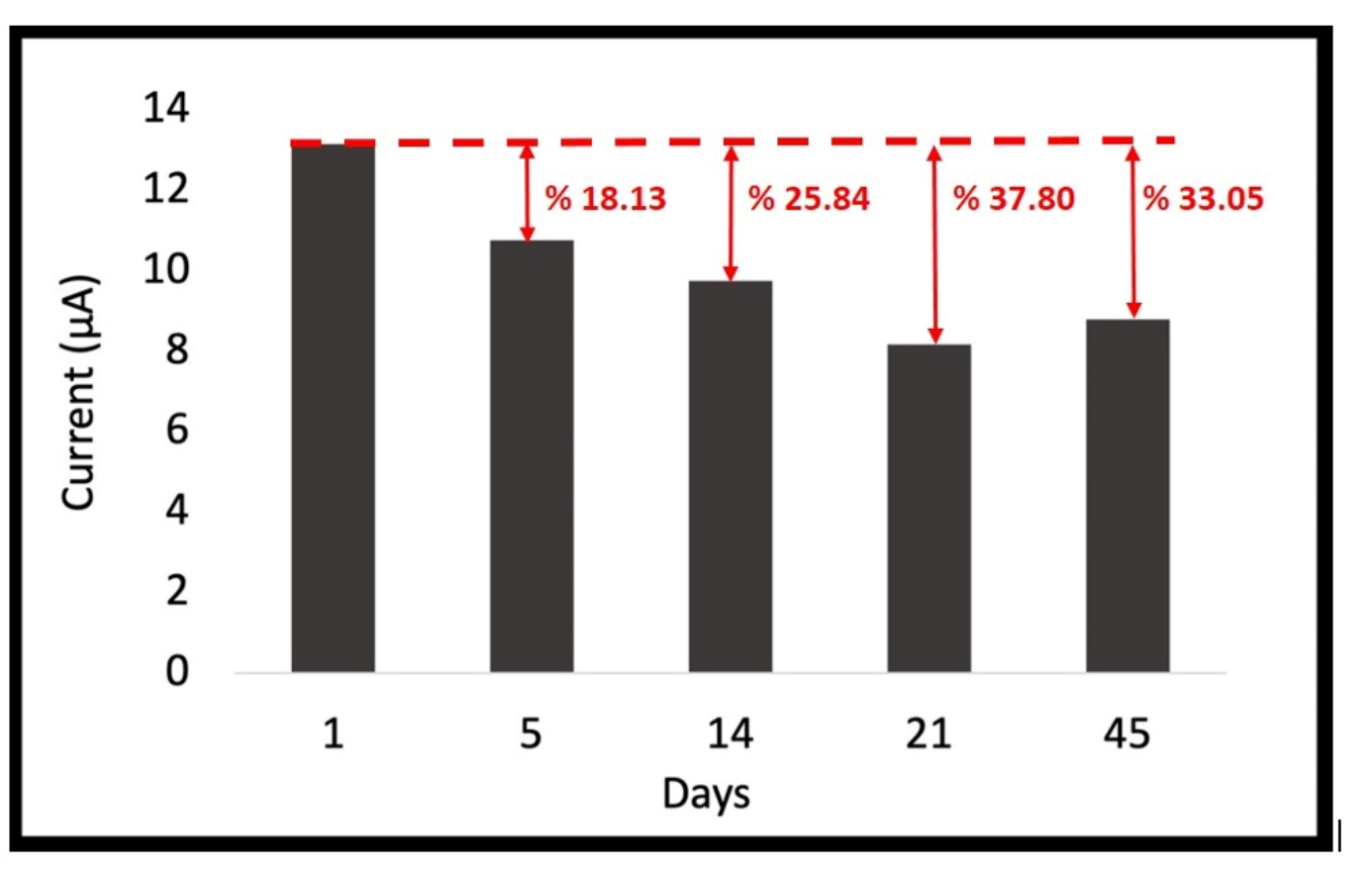


**Fig. S11.** Storage stability of the developed biosensor.

**Table S19.** DNA biosensors based on biopolymer-based composite materials.

| **Analyte** | **Electrode** | **Biopolymer** | **Electrode Preparation and Modification Time** | **Immobilization**  **Time** | **Measurement Method** | **Limit of Detection** | **Ref.** |
| --- | --- | --- | --- | --- | --- | --- | --- |
| DNA | LVN/PGE | Levan | 1 h 30 min | 1 h | DPV | 2.74 µg L^-1^ | [40] |
| ssDNA | SPE/KC-PPy-AuNPs | Carrageenan | - | 2 h | DPV | 5×10^−18^ M | [24] |
| DNA/dsDNA | Ag/CNs-dsDNA/GCE | Cellulose | 13 h | 1 h | DPASV | 2.3×10^−11^  mol L^-1^ | [64] |
| Mycobacterium tuberculosis  specific DNA | LSG-NF  AgNPs | Lignin | 3 h 20 min | 1 h | EIS | 1 fM | [65] |
| DNA/ dsDNA | PGE | CHIT | 2 h | 30 min | DPV | 1.14 μg mL^-1^ | [66] |
| DNA | MWCNT-CHIT  /SPCE | CHIT | - | - | CV | 16.7 μg mL^-1^ | [67] |
| fsDNA | CHIT-[GA-TiO_2_]/PGE | CHIT | 2 h 20 min | 15 min | DPV | 0.95  µg mL^-1^ | [10] |
| ssDNA | CMA/TEOS/PGE | Cellulose | 2 days | 20 min | DPV | - | [68] |
| fsDNA | 2B/PPy/CG-SA cryogel | Carrageenan and Sodium Alginate | 2 days | 30 min | CV | 0.98 µg mL^-1^ | This Study |

**ABBREVIATIONS: Modification:** LVN: Levan; PPy: Polypyrrole; AuNPs: Gold nanoparticles, CHIT: Chitosan; CN: Cellulose nanocrystal; Ag: Silver; LSG-NF: Green graphene nanofiber laser biosensor; AgNP: Silver nanoparticle; MWCNT: Multi-Walled Carbon Nanotube; CHIT-[GA-TiO_2_]: Chitosan-[Gallic Acid-Titanium Dioxide]; CMA/TEOS: Cellulose monoacetate/tetraethyl orthosilicate; CG-SA: Carrageenan and Sodium Alginate. **Method used:** DPV: Differential pulse voltammetry; DPASV: Differential pulse anodic stripping voltammetry; EIS: Electrochemical impedance spectroscopy. **Electrode used:** PGE: Pencil graphite electrode; GCE: Glassy carbon electrode; 2B: Pencil graphite electrode. **Analytes:** fsDNA: Fish sperm DNA; ssDNA: Single stranded DNA; dsDNA: Double stranded DNA.
